# Statistical learning abilities of children with dyslexia across three experimental paradigms

**DOI:** 10.1371/journal.pone.0220041

**Published:** 2019-08-05

**Authors:** Merel van Witteloostuijn, Paul Boersma, Frank Wijnen, Judith Rispens

**Affiliations:** 1 University of Amsterdam, Amsterdam Center for Language and Communication, Amsterdam, The Netherlands; 2 Utrecht University, Utrecht Institute of Linguistics OTS, Utrecht, The Netherlands; University of Haifa Faculty of Education, ISRAEL

## Abstract

Statistical learning (SL) difficulties have been suggested to contribute to the linguistic and non-linguistic problems observed in children with dyslexia. Indeed, studies have demonstrated that children with dyslexia experience problems with SL, but the extent of the problems is unclear. We aimed to examine the performance of children with and without dyslexia across three distinct paradigms using both on- and offline measures, thereby tapping into different aspects of SL. 100 children with and without dyslexia (aged 8–11, 50 per group) completed three SL tasks: serial reaction time (SRT), visual statistical learning (VSL), and auditory nonadjacent dependency learning (A-NADL). Learning was measured through online reaction times during exposure in all tasks, and through offline questions in the VSL and A-NADL tasks. We find significant learning effects in all three tasks, from which we conclude that, collapsing over groups, children are sensitive to the statistical structures presented in the SRT, VSL and A-NADL tasks. No significant interactions of learning effect with group were found in any of the tasks, so we cannot conclude whether or not children with dyslexia perform differently on the SL tasks than their TD peers. These results are discussed in light of the proposed SL deficit in dyslexia.

## Introduction

Dyslexia is one of the most common learning disabilities and is characterized by specific difficulties in learning to read and write despite normal intelligence, schooling and socio-economic opportunities and in absence of other impairments (e.g. sensory or neurological impairments [1]). These difficulties in the acquisition of literacy skills are typically associated with problems in related abilities including phonological awareness, lexical retrieval, and verbal short-term memory (e.g. [2–4]). For this reason, the predominant view of dyslexia is that the concomitant reading and writing problems stem from an underlying problem in the processing of phonological information (e.g. [4,5]). However, deficits in individuals with dyslexia may include other domains of language (e.g. inflectional morphology and syntax; [6,7]) and non-linguistic cognitive skills such as visual and auditory processing [8,9], attention [10] and motor functioning [11,12].

Due to this wide range of observed difficulties, it has been suggested that dyslexia is associated with a domain-general learning deficit rather than a deficit that is specific to the processing of phonological material (e.g. [13,14]). This domain-general learning mechanism is often referred to as *statistical learning* (SL): the ability to extract statistical regularities from sensory input [15], which is assumed to be a largely implicit process [16]. Importantly, SL is put forward as a key ability involved in the acquisition of language and literacy skills as it aids the discovery of the many rules and regularities that are present in spoken and written language [17]. In line with this reasoning and the hypothesized SL deficit in dyslexia, evidence shows that SL abilities are related to literacy skills in typical populations. For example, performance on tasks that assess SL abilities has been shown to positively correlate with reading in adults and children [18] and reading in a second language in adults [19]. Similarly, children with dyslexia have been shown to perform worse on tasks assessing SL abilities such as the Serial Reaction Time (SRT), Auditory Statistical Learning (ASL) and Artificial Grammar Learning (AGL) tasks (e.g. SRT [20,21]; ASL [22]; AGL [23]). However, others find no evidence of such an effect (e.g. SRT [24]; AGL [24,25]; cued reaction time task [26]). Literature reviews and meta-analyses have been conducted to investigate the overall group effect in SL studies and have reported significantly poorer performance by individuals with dyslexia as compared to those without dyslexia on both the SRT [27] and the AGL overall [28,29], although the effect on the AGL may be inflated due to publication bias in the field [28,29].

The current study aims to investigate to what extent children with dyslexia experience difficulties in the area of SL and to extend recent findings to other SL paradigms. It is important to study children specifically to clarify whether SL principles could potentially be used to improve treatment and clinical outcomes for individuals with dyslexia (see e.g. [30] on the clinical relevance of SL to children with developmental language disorder (DLD)). Since the hypothesized SL deficit has been claimed to be independent of the domain and modality in which SL is tested, children with dyslexia should experience difficulties across tasks tapping into SL abilities. Therefore, we assess children’s SL performance in a range of SL tasks that have previously been shown to be sensitive to learning in (typical) child populations and that span a number of methodological variations of SL tasks (e.g. modality, the type of statistical structure to be learned, online and offline measures): SRT, visual statistical learning (VSL), and auditory NADL (A-NADL) tasks. By measuring SL across different experimental paradigms using both online (SRT, VSL, A-NADL) and offline (VSL, A-NADL) measures, and by considering the potential differences in related cognitive abilities including memory and attention, we hope to provide a comprehensive study of SL abilities in children with dyslexia when compared to a control group of age-matched children. Before turning to the methodology of the present study, the following sections present an overview of previous studies investigating SL in dyslexia through the SRT, VSL and A-NADL paradigms. Subsequently, we discuss several methodological considerations that our design takes into account.

### Serial reaction time paradigm

The SRT task measures visuo-motoric sequence learning by exposing participants to a single visual stimulus that repeatedly appears in one of several locations on a computer screen [31]. Without the participants’ knowledge, the stimulus follows a predetermined order (i.e. sequence) over three or four locations. During exposure, participants are required to make motor responses that correspond to the locations of the individual stimuli on the screen. As the task unfolds, participants (implicitly) learn the repeated sequence of visual stimuli (locations in array), motor movements, or both, on the basis of the probabilities associated with the sequence. In other words, they learn the probability of the appearance of the stimulus in a given location on the basis of the locations of the previous trials. After participants have been repeatedly exposed to the sequence, they are unknowingly presented with a block of randomly ordered trials. An increase in reaction times (RTs) from predictable (i.e. sequences) to unpredictable (i.e. random) input during exposure is taken as evidence of sensitivity to the sequence presented to them [31]. A range of studies has demonstrated learning in the SRT both in TD adults and in TD children as young as 4 years of age (e.g. [32,33]).

The SRT task has frequently been used as a measure of statistical learning when investigating group differences between participants with and without dyslexia, both in adult (e.g. [34,35]) and child populations (e.g. [36,37]). The difference in sensitivity to SRT structure between participants with and without dyslexia was statistically significant in some studies (e.g. [38,39], the latter with 40 exposures) and not in others (e.g. [39–41], the first with 180 exposures). Lum et al. performed a meta-analysis of 14 such SRT studies involving both adults and children and showed that on average, non-dyslexic people outperform people with dyslexia ([27]; weighted average effect size = .449; *p* < .001). To summarize, the SRT task is known to be sensitive to learning in child populations and has been shown to differentiate between people with and without dyslexia.

### Visual statistical learning paradigm

Visual statistical learning (VSL) is a paradigm that assesses the capacity for SL by exposing participants to a continuous stream of visual stimuli such as abstract shapes (e.g. [42]) or cartoonlike figures (e.g. [18,43]). Unbeknownst to the participants, the stimuli in a VSL task are grouped together in groups of two (i.e. *pairs*) or three (i.e. *triplets*) that always appear together. This task is an adaptation of an auditory SL task that assesses word segmentation introduced by Saffran, Aslin and Newport [44]. Thus, in the VSL, the probability of one stimulus following the preceding one differs per trial: while the second (and third) stimulus within a pair (and triplet) is predictable, the first stimulus of the next group is unpredictable. Following repeated exposure to the structured stimuli, a test phase assesses the participants’ ability to distinguish previously seen groups of stimuli from groups of stimuli that did not co-occur frequently during exposure. By applying this experimental paradigm, it has been shown that not only adults show sensitivity to this type of statistical structure (e.g. [42,43,45]), but also school-aged children [18,43,46]), as well as infants when tested in a preferential looking time paradigm (e.g. [47]). Similar results have been reported for studies involving auditory stimuli including syllables (e.g. [44]) or non-verbal stimuli such as tones (e.g. [48]).

Relevant to the present investigation, only two previous studies have examined the SL abilities of participants with dyslexia using a variant of the VSL task [49,50]. In a study by Sigurdardottir et al. [49], the exposure phase comprised twelve abstract visual shapes that were divided into six pairs of co-occurring stimuli, and participants were subsequently tested in a two-alternative forced-choice (2-AFC) test phase consisting of 72 trials. The results show that adult participants with dyslexia reached lower accuracy levels in the test phase than the control group in the VSL task (68% vs. 78% respectively). The second study investigated the event-related potential (ERP) correlates of SL in children with and without dyslexia using a visual task [50]. During the task, children were continuously exposed to series of colored circles and were required to respond to a target color through a button press. Although RT data revealed no difference between children with and without dyslexia (N = 8 and 12 respectively), ERP data reveal indications of learning in the control group, but not in participants with dyslexia. Although these studies suggest poorer sensitivity to VSL structures in participants with dyslexia as compared to control participants, no study to date has applied the standard ‘triplet’ paradigm (e.g. [18,43,45]) to children with dyslexia. Moreover, no data regarding explicit judgments of VSL structure is available on children with dyslexia.

### Nonadjacent dependency learning paradigm

Gómez [51] aimed to test learning of a different type of structure: nonadjacent dependencies. In this type of structure, participants learn relationships between nonadjacent elements, ignoring variable intervening elements; for instance, in the string *aXb*, *a* predicts *b* and *X* is a variable intervening element. This experimental design relates to nonadjacent dependencies found in natural language, such as those in inflectional morphology (e.g. *is* eat*ing*, *has* eat*en*, where the auxiliary predicts the inflectional morpheme regardless of the intervening verb; [51,52]). Not only adults, but also infants at age 1;6 were sensitive to such nonadjacent dependencies through mere exposure when 24 intervening *X*-elements are used. This is reflected by differences in responses when, after the exposure phase, they are confronted with strings that adhere to the *aXb* grammar as opposed to strings that do not (e.g. *aXc*; [51]). However, not much is known about the performance of school-aged children on tasks involving nonadjacent relationships. One previous study has investigated NADL in children using the Gómez [51] design and reports above-chance performance on grammatical items in TD children, suggesting sensitivity to the NADL structure [53].

The same paradigm was used to investigate sensitivity to non-adjacent dependencies in relation to dyslexia. Kerkhof et al. [54] tested infants with and without a family risk of dyslexia around the age of 1;6 on a slightly adapted version of the NADL task containing two nonadjacent dependencies of the type *aXb* [51]. In the subsequent test phase that consisted of 8 trials, results reveal a significant interaction between grammaticality and risk group: infants without family risk are sensitive to the NADL structure (i.e. they listen longer to ungrammatical than grammatical strings), while infants at risk of dyslexia are less sensitive, if at all. A follow-up study from the same lab examined NADL in the auditory and visual domain in Dutch-speaking adults with and without dyslexia [55]: participants were tested on two versions of the auditory experiment containing either test sentences with familiar *X*-elements or test sentences with novel *X*-elements that aimed to test generalization of the rule. On average, participants were more likely to accept (i.e. endorse) grammatical than ungrammatical sentences in both conditions, reflecting sensitivity to the nonadjacent dependency rule, but no interaction was detected between this measure of learning and group. Similar results are reported for NADL by adults in the visual domain. To summarize, differences in sensitivity to the A-NADL structure were found in infants with and without risk of developing dyslexia, and the results for adults are inconclusive. To our knowledge, no reports of school-aged children with dyslexia on tasks assessing NADL have been published.

### The current study

A number of methodological considerations become apparent from previous literature that are relevant for our investigation of SL in dyslexia. Firstly, and perhaps most importantly, the majority of studies has focused on infant and adult participants. Whereas the SRT and AGL tasks have been used in child populations with and without dyslexia, studies employing alternative paradigms such as the VSL and NADL have not been used to investigate SL in school-aged children with dyslexia.

Secondly, although SL is thought to be a domain-general learning mechanism, task parameters and participant characteristics are likely to influence the magnitude of the learning effect found in individual studies [15,45]. Researchers have previously emphasized the importance of using a range of SL measures within a single sample when investigating the hypothesized SL deficit in children, as opposed to using only one SL paradigm as is common in most studies [56,57].

Thirdly, VSL and NADL tasks have commonly used offline measures to assess learning after exposure. While these measures inform us about the *outcome* of the learning process, they do not inform us about the learning process itself [58–61]. Recently, two studies have introduced child-friendly VSL [62] and A-NADL [63] tasks that include online measures of learning adapted from previous studies with adult participants [60,64]. These online measures reflect participants’ sensitivity to statistical regularities during exposure to the stimuli and may provide further insights into the potential differences in performance between children with and without dyslexia when used in addition to the more traditional offline measures.

Finally, studies have shown that performance in SL tasks is affected by cognitive abilities such as attention (e.g. [65,66]). Arciuli [67] has argued that SL is not only related to attention but may also partly rely on (short-term, working and long-term) memory (see also [43,68,69]). Important to the present discussion is the fact that individuals with dyslexia have difficulties in the area of attention (e.g. [70,71]) and short-term and working memory (e.g. [72]).

The present study aims to address the abovementioned methodological considerations by assessing the performance of children with and without dyslexia on three different experimental paradigms using a range of online (SRT, VSL and A-NADL) and offline (VSL and A-NADL) measures. In doing so, we want to provide a comprehensive study in which we investigate to what extent children with dyslexia have difficulty in SL. In all analyses, we address two research questions:

1. Do we find evidence of sensitivity to the statistical structure in the SRT, VSL and A-NADL tasks in children overall?
2. Do we find evidence of a difference in performance on the SRT, VSL and A-NADL tasks between children with and without dyslexia?

If children with dyslexia experience general difficulties with SL, we expect to find group differences across the different tasks tapping into SL regardless of the characteristics of the task (e.g. domain, modality or type of structure to be learned). By running subsequent exploratory analyses that control for sustained attention and visual and auditory short-term and working memory, we take into account the possibility that potential group differences in SL are due to underlying differences in these cognitive abilities (i.e. do children with dyslexia experience problems with SL independent of potential difficulties with sustained attention and shot-term and working memory?). Thus, the present study will shed light on the mechanisms underlying the reading problems experienced by individuals with dyslexia: could a domain-general deficit in SL contribute to these problems?

## Materials and methods

### Participants

Participants in the present study were tested as part of a larger study that investigates SL and its relationship with language skills in children with dyslexia, children with DLD and TD children (e.g. [62,63]). Ten out of 60 participants with a prior formal diagnosis of dyslexia were excluded because they did not meet our pre-determined inclusion criterion of scoring an average of 6 or less (the 10th percentile) on word reading and nonword reading. Similarly, 4 out of 54 children in the TD group were removed for not meeting our inclusion criterion of scoring an average of 8 or more (the 25^th^ percentile). Consequently, the final sample consisted of 50 children with dyslexia (26 girls, 24 boys, age range 8;4–11;2, *M* = 9;10) and 50 age-matched TD children (24 girls, 26 boys, age range 8;3–11;2, *M* = 9;8). None of the children had diagnoses of (additional) developmental disorders and all children were native speakers of Dutch (at least one parent spoke Dutch at home) and were reported to have IQ levels within the normal range of the general population. Group characteristics, including raw and standardized scores on several background measures, are presented in Table 1.

**Table 1:** Minimum, maximum and mean (*SD*) raw and standardized scores on background measures and measures assessing memory and sustained attention per group, including group comparison statistics.

	Dyslexia (*N* = 50)		Control (*N* = 50)	
	Raw	Standardized	Raw	Standardized
Age	8;4–11;2 9;10 (0;9)	N/A	8;3–11;2 9;8 (0;10)	N/A
SES	-3.31–2.09 0.2 (1.2)	N/A	-1.28–1.41 0.2 (1.1)	N/A
Nonverbal reasoning^a^	23–49 37.2 (6.6)	7–95 55.7 (25.0)	16–55 37.3 (8.1)	6–98 60.1 (28.1)
Reading words^b^	8–59 34.1 (11.7)	1–7 3.3 (2.1)	44–92 66.3 (11.6)	7–15 10.5 (2.2)
Reading pseudo-words^b^	8–39 22.0 (8.0)	1–7 4.4 (1.6)	33–89 61.0 (14.4)	7–15 11.1 (2.2)
Spelling^a^	0–17 8.4 (4.6)	0–71 11.8 (13.7)	9–27 18.6 (4.7)	6–95 49.9 (24.7)
RAN pictures^b^	35–80 53.2 (10.2)	2–14 7.7 (2.7)	30–63 44.1 (7.3)	5–16 10.7 (2.8)
RAN letters^b^	23–79 36.1 (10.4)	1–12 5.4 (2.7)	18–46 27.2 (5.5)	3–16 9.6 (3.1)
Sustained attention^b^	1–10 7.0 (2.5)	1–13 7.4 (3.3)	3–10 7.8 (1.8)	3–14 9.1 (3.0)
Digit span forward^b^	4–11 7.3 (1.5)	1–13 7.7 (2.6)	6–12 8.9 (1.5)	5–15 10.7 (2.9)
Digit span backward^b^	2–7 4.2 (1.1)	1–14 9.0 (2.5)	2–8 4.5 (1.5)	4–16 10.0 (3.2)
Dot matrix forward^cd^	15–35 25.1 (4.7)	N/A	13–34 25.7 (5.1)	N/A
Dot matrix backward^cd^	8–35 22.9 (5.0)	N/A	15–34 24.1 (4.9)	N/A

*Note*: Raw scores represent the number of items answered correctly out of 60 on the Raven, the number words and pseudo-words read correctly within 1 minute and 2 minutes respectively, the number of words spelled correctly out of 30, the number of seconds spent on the task in case of the RAN (i.e. higher score = lower performance), the number of items answered correctly on sustained attention (max = 10) and subtests of the digit span (max = 16) and the dot matrix (max = 36, following the AWMA scoring procedure). Standardized scores represent either ^a^ percentile scores (norm = 50) or ^b^ norm scores (norm = 10). For the dot matrix task, ^c^ standardized scores are unavailable and ^d^ data is based on 49 children with dyslexia, due to missing data for one participant as a result of equipment failure.

Children with dyslexia were recruited through treatment centers in Amsterdam (*N* = 25) and Amersfoort (*N* = 10) and through parent support groups on Facebook (*N* = 11). Four children with dyslexia were tested along with the control group in four schools across the province of Noord-Holland in the Netherlands. The ethical committee of the University of Amsterdam approved the protocol for the present study in 2016. All parents and/or legal guardians of participants were informed about the project through a newsletter. Compliant with the regulations of the ethics committee, informed consent was obtained from the parents and/or legal guardians of children with dyslexia prior to testing (active consent). For the control group, schools and teachers consented to participation, and parents and/or legal guardians could retract permission of including their child up to 8 days following testing (passive consent).

To compare the group of participants with dyslexia with their TD peers on the range of included background measures, we fitted linear models on the raw data using the *lm* function for R software [73]. No significant differences were found between the chronological ages of the groups (*t* = 0.839, *p* = .40), the groups’ socio-economic status (SES; *t* = 0.173, *p* = .86) or non-verbal reasoning (*t* = -0.041, *p* = .97). SES scores were obtained from the *Netherlands Institute for Social Research* (NISR) on the basis of children’s home or school postal codes depending on the testing location. These SES scores were calculated by the NISR in 2017 and indicate the social status of a given neighborhood in comparison to other neighborhoods in the Netherlands (open source data can be accessed through the following (Dutch) link: https://www.scp.nl/Onderzoek/Lopend_onderzoek/A_Z_alle_lopende_onderzoeken/Statusscores). Non-verbal reasoning was assessed through *Raven’s Standard Progressive Matrices* [74]. We also measured children’s reading of single Dutch words (*Een Minuut Test*; [75]) and pseudo-words (*Klepel*; [76]), their spelling (*Schoolvaardigheidstoets Spelling*; [77]) and their rapid automatized naming (RAN) of pictures and letters (*Continu Benoemen en Woorden Lezen*; [78]). In line with expectations, analyses show that children with dyslexia performed significantly moor poorly than the TD children on all measures assessing literacy skills (reading words: *t* = -13.83, *p* = 9·10^−25^, reading pseudo-words: *t* = -16.75, *p* = 1.7·10^−30^, spelling: *t* = -11.42, *p* = 9.4·10^−20^, RAN pictures and letters: *t* = -4.985, *p* = 2.7·10^−6^ and *t* = -5.421, *p* = 4.3·10^−7^ respectively).

We assessed cognitive abilities that are often associated with SL and that may differ between our groups of participants with and without dyslexia: short-term and working memory and attention (see Table 1). Short-term and working memory were tested in the auditory domain with the forward and backward digit span tasks from the Dutch version of the *Clinical Evaluation of Language Fundamentals* [79] and using forward and backward versions of the dot matrix task in the visuospatial domain [80]. Sustained attention was measured through the *Score*! subtest of the Dutch *Test of Everyday Attention for Children* [81]. In this task, children perform 10 items that contain between 9 and 15 target sounds that are presented at varied intervals. Their task is to silently count the target sounds, reflecting the child’s ability to maintain attention over time. The digit span backward and dot matrix forward and backward did not reveal significant differences between participants with and without dyslexia (digit span backward: *t* = -1.257, *p* = .21, dot matrix forward: *t* = -0.667, *p* = .51, dot matrix backward: *t* = -1.248, *p* = .22). Digit span forward performance (i.e. verbal short-term memory) was significantly poorer in participants with dyslexia as compared to their TD peers (*t* = -5.36, *p* = 5.5·10^−7^). The groups differed marginally significantly in sustained attention (*t* = -1.939, *p* = .055). Given these findings, we explore whether adding the digit span forward and sustained attention scores to our models influences our findings regarding SL performance (see section on scoring and analysis).

### SRT task

A visual stimulus (yellow smiley face) repeatedly appeared in one out of four marked locations on a black background presented on a tablet screen. Participants were instructed to press corresponding buttons on a gamepad as quickly and accurately as possible and practiced the task in 28 trials. Each instance of the visual stimulus was visible until a response was given, with a 250 milliseconds interval before the next instance of the stimulus appeared. Participants had a maximum of 3 seconds to respond before the task would move on to the next instantiation of the stimulus automatically.

Unbeknownst to the participant, the stream of stimuli was divided into seven underlying blocks. The first block contained 20 random trials. Blocks 2 through 5 and block 7 contained structured input that consisted of six repetitions of a 10-item sequence (i.e. sequence blocks, 60 trials each). The sequence consisted of a constant order of locations (quadrants) in which the visual stimulus appeared (quadrants 4, 2, 3, 1, 2, 4, 3, 1, 4, 3). In disruption block 6, the appearances of the stimulus no longer followed the sequence, but was presented in random order (i.e. 60 random trials). Both accuracy and RT to each stimulus presentation were recorded. If learning takes place in the SRT task, RTs to predictable input averaged over sequence blocks 5 and 7 are expected to be shorter than RTs to unpredictable input in the intervening disruption block [31]. The SRT task in the present study did not include an explicit offline test phase.

### VSL task

#### VSL task: Online exposure phase

In the VSL task, visual stimuli were presented one at a time in the middle of a tablet screen. Without the participants’ knowledge, stimuli appeared in the same four groups of three (i.e. triplets; *ABC*, *DEF*, *GHI*, and *JKL*). The exposure phase of the VSL task is divided into four blocks containing six repetitions per triplet, resulting in 24 repetitions of each triplet. Following previous studies adopting a similar structure [18,42,43], triplets could not appear twice in a row and pairs of triplets could not be repeated (i.e. sequences such as *ABC*, *ABC* or *ABC*, *JKL*, *ABC*, *JKL* could not occur). The VSL structure can be expressed in terms of predictability through the transitional probabilities (TPs; the probability of event *i*+1 given event *i*): given the occurrence of element *A*, the TP to element *B* is 1 and the same holds for element *C* given element *B*. The TP when crossing a triplet boundary is low. Thus, whereas elements 2 and 3 within triplets (e.g. stimuli *B* and *C* in the triplet *ABC*) are completely predictable, the first element of the following triplet (e.g. stimulus *D* of the triplet *DEF*) is less predictable.

The self-paced nature of the task entails that participants responded to each individual stimulus by pressing the space bar, upon which the next stimulus appeared after 200 milliseconds [60,62]. We recorded RTs to individual stimuli, which were used as an online measure of learning. If learning takes place, RTs to predictable stimuli (i.e. elements 2 and 3 within triplets) are expected to be shorter than RTs to unpredictable stimuli (i.e. element 1 within triplets). Thus, learning in the online phase of the VSL is reflected by a difference in RTs to predictable as compared to unpredictable stimuli, since sensitivity to the statistical structure is hypothesized to result in faster processing of predictable stimuli (as in the SRT task).

As part of the cover task, three stimuli per block were presented twice in succession (i.e. 12 repetitions in total; [18,43]). In the event of a repeated stimulus, participants were required to respond by tapping the alien on the touch screen. Each triplet contained a double stimulus three times throughout the exposure phase, all three elements within the triplet once (e.g. the triplet *ABC* occurs once as *AABC*, *ABBC*, and *ABCC*). In each block, three distinct triplets contained a double stimulus in random positions of the stream of stimuli, again all three element positions within triplets once (e.g. *AABC*, *DEEF*, *GHII*).

#### VSL task: Offline test phase

To test participants on their acquired knowledge of the triplet structure, they were tested in an offline test phase subsequent to exposure that consisted of 40 multiple-choice questions. Using the same set of 12 stimuli as used in the familiarization phase, four foil triplets were created (*AEI*, *DHL*, *GKC*, and *JBF*, all with TPs of 0 within triplets). Participants first received three-alternative forced choice (3-AFC) questions in which they were asked to complete a missing shape (*N* = 16, chance level = .333) and subsequently questions in which they were required to pick the more familiar pattern out of two options (2-AFC; *N* = 24, chance level = .500). Test items either tested complete triplets (3-AFC: N = 8, 2-AFC: N = 8) or pairs within triplets (3-AFC: N = 8, 2-AFC: N = 16). Learning in the VSL test phase is evidenced by above-chance performance, since above-chance performance reflects participants’ ability to explicitly judge which patterns belong to the statistical structure in the VSL task.

#### VSL task: Procedures

Importantly, the effect of single stimuli or triplets and the effect of the order of appearance during familiarization and testing were counter-balanced: two sets of triplets (and foil triplets) were created using the same set of 12 stimuli, and two random orders of the presentations of triplets during exposure and testing were created. This resulted in four versions of the experiment, to which participants were randomly assigned.

Before the exposure phase, participants performed two practice phases consisting of an alternative set of stimuli. In the first practice phase, participants practiced sending home the aliens by pressing the space bar (N = 16). In the second part, children were instructed to pay attention to double stimuli and instructed to tap the touch screen in these cases (N = 18, 3 double stimuli). Importantly, children were instructed to pay attention to the aliens and were informed that some of the aliens liked each other and stood in line together. In between the four blocks of the experiment, participants received stickers for a diploma and were stimulated to pay attention to the aliens. Prior to each of the two parts of the offline test phase, participants received instructions and a practice trial during which they were encouraged to make a guess in case they were unsure of the correct response.

### A-NADL task

#### A-NADL task: Online exposure phase

Children were exposed to an artificial language that, unbeknownst to them, contained two nonadjacent dependencies in 80% of the trials: *tep X lut* and *sot X mip*, where *tep* predicted *lut* and *sot* predicted *mip* and the variable intervening X-element always consisted of two syllables (e.g. *wadim*, N = 24; e.g. [51]). The remaining 20% of the trials were filler trials that deviated from the two nonadjacent dependencies. These trials can be described as *fXf* trials and resembled the *aXb* nonadjacent dependency structure: the elements in the *f* positions consisted of one-syllable nonwords (*N* = 24) and were separated by the same X-elements used in the nonadjacent dependencies. In filler trials, however, the first *f* element did not predict the second *f* element. Each trial in the A-NADL thus consisted of three elements and was between 2067 and 2908 milliseconds long (*M* = 2415 milliseconds) with an interval of 250 milliseconds between elements. All stimuli used in the A-NADL were created in accordance with Dutch phonotactic constraints, followed a natural Dutch sentence prosody (e.g. *het meisje loopt*, the girl walks), and were recorded by a female native speaker of Dutch.

The task consisted of a total of 270 trials divided into five blocks: four blocks containing the nonadjacent dependency rules and fillers (rule blocks 1–3 and 5) and one intervening block in which the strings did not contain the nonadjacent dependency rules (disruption block 4). Forty-eight trials in each of the rule blocks contained the two nonadjacent dependencies (24 times *tep X lut* and 24 times *sot X mip*) in addition to 12 fillers, resulting in a total of 60 trials per rule block. Both nonadjacent dependencies were presented in combination with each X-element once in each block and thus repeated four times during the exposure phase (i.e. 96 exposures to each nonadjacent dependency). The disruption block contained 30 trials in which the rule structure was disrupted: trials were of the structure *f X lut* and *f X mip*, so that the occurrences of *lut* and *mip* were no longer predictable (*N* = 12 each). The remaining six trials were filler items. Combinations of filler elements (*f*) and X-elements were unique and only appeared once across the duration of the exposure phase (N = 54).

Importantly, the online measure of learning was a word-monitoring task that required participants to attend to the speech stream and track the occurrence of a *target* (i.e. a specific nonword) and respond as quickly as possible by pressing a button on an external button box [63,64]. The target was always the predictable *b* element of one of the two nonadjacent dependencies (i.e. *lut* or *mip*) and participants were randomly assigned to one of two experiment versions (version 1: target = *lut*, version 2: target = *mip*). Predictable element *b* of the unattended nonadjacent dependency will henceforth be referred to as the *nontarget* (version 1: nontarget = *mip*, version 2: nontarget = *lut*). When the trial contained the target, participants were required to press the green button, while they were required to press the red button when the trial did not contain the target. For example, in version 1 of the experiment, where *lut* was the target, participants had to press the green button when trials contained the target word *lut* (rule blocks: *tep X lut*, disruption block: *f X lut*) and press the red button when trials contained the nontarget word *mip* (rule blocks: *sot X mip*, disruption block: *f X mip*) or contained neither *lut* or *mip* as was the case in filler items. Children had 1500 milliseconds to respond to each trial before the experiment moved on to the next trial automatically. Accuracy and RT were recorded for each individual trial.

As in the SRT and VS tasks, learning in the online measure of the A-NADL is defined as the difference in RTs between predictable and unpredictable input. The target and non-target words were predictable during rule blocks (they were always preceded by the corresponding *a* element as in *tep X lut* and *sot X mip*) but were no longer predictable in the disruption block (they were no longer preceded by the *a* element but by a variable *f* element as in *f X lut* and *f X mip*). Thus, mimicking the structure of the SRT task, learning in the A-NADL task is evidenced by shorter RTs to both target and nontarget trials in rule blocks 3 and 5 as opposed to the intervening disruption block.

#### A-NADL task: Offline test phase

Participants were tested on their acquired knowledge of the nonadjacent structure through offline grammaticality judgments (N = 16). They were required to indicate whether they had previously heard each string by saying either ‘yes’ (endorsement) or ‘no’ (rejection). Eight items were grammatical strings (e.g. *sot densim mip*) and eight were ungrammatical strings where the nonadjacent dependency structure was disrupted (e.g. *sot filka lut*). Similarly, eight strings contained familiar X-elements used during exposure and eight strings contained novel X-elements that were only used during the test phase. Two additional items that contained three X-elements (i.e. *XXX*) functioned as filler items and were not included in analysis. If learning in the test phase of the A-NADL is successful, we expect to find a higher proportion of endorsements as opposed to rejections to grammatical strings than to ungrammatical strings.

#### A-NADL task: Procedure

There were two counterbalancing variables in the A-NADL: children either received a version of the task where the target was *lut* or the target was *mip* and the location of the green and red buttons on the external button box were counter-balanced. Participants were randomly assigned to the four versions of the experiment.

Participants were seated behind a tablet and held the button box in their hands, using both thumbs to press the buttons. The auditory stimuli were played through headphones. The word-monitoring task was framed as a game in which the participant helped a monkey to pick bananas. Children were told that they would hear three-word sentences and had to press the green button when they heard the target and the red button when they did not. In accurate trials, the monkey was rewarded with a banana. Children were instructed to pay attention to all three words in the sentences, since they would receive questions at the end. A practice phase containing six items preceded the start of the experiment, which was repeated until they reached a score of 4 out of 6 correct (for which they had to master the motorics and press in time). During the exposure phase, the experiment was broken up into short blocks containing 30 trials each. Following these blocks, children received feedback on the number of bananas they picked and received a sticker for their diploma. Subsequent to exposure, the experimenter instructed the participant that they would hear sentences one at a time and to indicate whether they had heard the sentence before or not. Two practice items preceded the GJT and children were encouraged to guess if they were uncertain of the answer.

### General procedure

All SL tasks were programmed and ran using E-prime 2.0 software [82,83] on a Windows Surface 3 tablet with touchscreen and keyboard. Auditory instructions (and stimuli in the case of the A-NADL) were played over Sennheiser HD 201 headphones. Additional materials included the gamepad used in the SRT task (Trust wired gamepad GXT540) and the external button box used in the A-NADL task.

As mentioned previously, participants in the present study were tested as part of a larger study. Children were tested individually by an experimenter in a quiet room either at home or at school. Testing lasted approximately three hours, divided over three testing sessions that lasted around an hour. In each of these sessions, one of the SL tasks was administered along with three or four of our linguistic or cognitive measures (each of these was measured only once). The order of the sessions (and the order of tasks within sessions) was counter-balanced: six testing orders were created to which participants were assigned randomly. Thus, the order of the SL tasks (order 1: A-NADL, SRT, VSL; order 2: SRT, VSL, A-NADL; order 3: VSL, A-NADL, SRT), as well as the linguistic and cognitive measures, was semi-randomized. Each child was rewarded for their participation with stickers on a diploma and a small present after completing the three sessions.

### Scoring and analyses

Online RT data of the SRT, VSL and A-NADL tasks was analyzed with linear mixed effects models that were built using the *lme4* package (version 1.1–13; [84]) for *R* software [73]. Similarly, the *lme4* package for *R* was used to build generalized linear mixed effects models for the offline accuracy data in the VSL and A-NADL tasks. Wherever possible, a confidence interval (CI) was computed by the profile method (*stats* package version 3.5.2 for *R* software; [73]), and a corresponding p-value was obtained by interpolation among the profiles for different CI criteria (e.g. a p-value of .03 was concluded if one of the edges of the 97 percent CI was zero; see “get.p.value” function on OSF). In the A-NADL offline measure, some CIs were computed using Wald’s approximation for CI’s and *p*-values are obtained from the model output. This was only done when (1) the profile function failed to provide CIs, *and* (2) we did not want to further decrease the random effects structure, *and* (3) the result was non-significant. For all analyses, continuous predictors were centered and scaled, while categorical predictors were coded into orthogonal contrasts. Group is always orthogonally coded such that the control group is marked as -^1^/_2_ and the dyslexia group is marked as +^1^/_2_. Therefore, the effect of group is always interpreted as the change in effect when moving from the control group to the group of participants with dyslexia (see Table 2 for an overview of all orthogonally coded categorical predictors per SL task and following sections for further explanation). In line with Barr, Levy, Scheepers and Tily [85], models contained the maximal random effect structure, unless this resulted in a failure to fit the model or in (near-)perfect correlations between the random effects in which case reductions were performed that are explicitly justified in the text. Raw data and *R* Markdown and html files detailing all analyses of the SRT, VSL and NADL tasks can be accessed through the following link: https://osf.io/t8scv/?view_only=eb217175b3cc4d5ebd4d3e4549ada64f.

**Table 2:** Orthogonal contrast coding of categorical predictors in the SRT, VSL and A-NADL tasks.

Task	Predictor	Contrast coding	Purpose
SRT	Block (Bl)	1: Bl 6 = -^2^/_3_, Bl 5 and 7 = +^1^/_3_	RQ 1
		2: Bl 5 = -^1^/_2_, Bl 7 = +^1^/_2_	Exploratory
	Group	TD = -^1^/_2_, DD +^1^/_2_	RQ 2
VSL	Element (El)	1: El 1 = -^2^/_3_, El 2 and 3 = +^1^/_3_	RQ 1
		2: El 2 = -^1^/_2_, El 3 = +^1^/_2_	Exploratory
	Group	TD = -^1^/_2_, DD +^1^/_2_	RQ 2
	Triplet Set (TS)	TS A = -^1^/_2_, TS B = +^1^/_2_	Exploratory
	Random Order (RO)	RO 1 = -^1^/_2_, RO 2 = +^1^/_2_	Exploratory
A-NADL	Block (Bl)	1: Bl 4 = -^2^/_3_, Bl 3 and 5 = +^1^/_3_	RQ 1
		2: Bl 3 = -^1^/_2_, Bl 5 = +^1^/_2_	Exploratory
	Grammatical	No = -^1^/_2_, Yes = +^1^/_2_	RQ 1
	Group	TD = -^1^/_2_, DD +^1^/_2_	RQ 2
	Generalization	No = -^1^/_2_, Yes = +^1^/_2_	Exploratory
	Target Type	Target = -^1^/_2_, Non-target = +^1^/_2_	Exploratory
	Experiment Version	Lut = -^1^/_2_, Mip = +^1^/_2_	Exploratory

*Note*: RQ = research question: RQ 1 pertains to the overall learning effect, while RQ 2 regards the effect of group (dyslexia versus control) when looked at in interaction with the effect of learning overall. Exploratory predictors and contrasts are included either because predictors are counter-balancing factors or because predictors need to be orthogonally coded (i.e. in the case of predictors with two contrast codings).

For online RT measures, analyses were first run on the raw data. However, in all three tasks, this resulted in non-normally distributed residuals of the linear mixed effects models as evidenced by their QQ-plots. Therefore, we decided to use a rank order transformation, which is a principle non-arbitrary way to reduce the effect of outliers and to reduce skewness in the distribution of the residuals (see [86], p. 354–358). The commonly used log-transformation was not appropriate due to the presence of negative RTs. The rank-order transformation is was done by ranking the *N* pieces of pooled data from 1 to *N*, then computing the inverse cumulative Gaussian distribution (with the following formula in *R*: qnorm((ranking-0.5)/*N*)); the model estimates hereby come to represent differences in *z*-values (*Δz*; e.g. the main effect of a binary predictor is given by the change in *z*-value from one level to the other).

As part of our exploratory findings, we compute additional models for each of our SL measures to investigate the effect of adding sustained attention and verbal short-term memory as continuous predictors; the fit of each of these models is compared statistically to the model without these two measures. At the request of reviewers, children’s chronological age was added as an exploratory (continuous) predictor in all models. This was done to reduce variance and to examine whether age interacts with the measures of learning in the SL measures (relating to research question one) and group (relating to research question two). Only significant findings regarding the exploratory effect of age are included in the results section. The subsequent sections provide further details regarding the pre-processing of the data and the analyses of the three SL tasks.

#### SRT task

The first block of the SRT, containing 20 random presentations of stimuli, was removed from analysis. Furthermore, incorrect responses and trials in which no response was given were removed from the data file (5.9% data loss).

The linear mixed effects model was run using normalized RTs as the dependent variable. Since online sensitivity to the sequence in the SRT task is measured as the difference in RTs to predictable versus unpredictable input, our analysis contrasted RTs in sequence blocks with RTs in the intervening disruption block that contained random input in order to answer research question one (i.e. within-participant predictor Block: block 5 and 7 vs. block 6). The categorical predictor Block was orthogonally coded into two contrasts: the effect of learning (i.e. random block 6 coded as -^2^/_3_ vs. sequence blocks 5 and 7 coded as ^+1^/_3_ each, thereby comparing random block 6 to the average of sequence blocks 5 and 7) and the contrast between the two sequence blocks (block 5 vs. block 7 coded as -^1^/_2_ and +^1^/_2_ respectively). Further predictors in the model included the between-participants predictors Group (control versus dyslexia) and Age. To answer our second research question, we looked at the interaction between the first level of Block and Group. The model included by-subject random intercepts and by-subject random slopes for Block.

#### VSL task

Our scoring and analysis procedures of the online RT measure followed those by Van Witteloostuijn et al. [61]. The RTs to the first triplet in each block of the experiment were removed (4.2% data loss). This was done because these responses are likely to deviate from participants’ normal patterns. Additionally, RTs shorter than 50 milliseconds were removed from the dataset, as these were assumed to reflect cases in which the participant did not process the stimulus (0.2% data loss).

Sensitivity to the structure is measured as the difference in RT to unpredictable versus predictable elements within triplets; this sensitivity may depend on time (research question one). Thus, the model fitted normalized RTs as a function of the within-participant predictors Element (element 1, 2 and 3 within triplets) and Time (repetitions 1–24 of triplets). The categorical predictor Element was orthogonally coded into two contrasts: the effect of learning (i.e. element 1 coded as -^2^/_3_ vs. element 2 and 3 coded as +^1^/_3_ each), and the contrast between the two predictable elements (element 2 coded as -^1^/_2_ and element 3 coded as +^1^/_2_). The interaction between the effect of learning (i.e. the first level of Element) and the between-participant predictor Group (control versus dyslexia), and its three-way interaction with Group and Time were of interest to our second research question. Two counter-balancing factors were included in the model as within-participant predictors (Triplet Set A and B coded as -^1^/_2_ and +^1^/_2_ respectively and Random Order version 1 and 2 also coded as -^1^/_2_ and +^1^/_2_ respectively). Finally, the model contained the exploratory between-participants predictor Age. The random effect structure included by-subject and by-item intercepts, as well as by-subject random slopes for Element and Time and the interaction between the two. The individual aliens used in the experiment (*N* = 12) were used for the random intercepts for item. By-item random slopes for group were removed, since these resulted in a perfect correlation between the random intercept for item and the by-item random slopes for group (i.e. the model was overparameterized). This removal did not result in a decrease in the fit of the model (*χ*^*2*^ = 0.0655, *df* = 2, *p* = .968). In order to compute the CIs and p-values of the final model, the interaction between Element and Time, which was non-significant, was removed from the random effects structure.

In the offline test phase, responses were coded as either correct or incorrect (i.e. 1 or 0). Accuracy is expressed as the proportion of correct responses, such that chance levels are .333 and .500 for the 3-AFC and 2-AFC questions respectively. No accuracy data was removed prior to running the generalized linear mixed effects models.

Two models were constructed to analyze the 3-AFC and 2-AFC accuracy data separately. To answer our first research question as to whether learning took place, we examined whether the proportion of accurate responses exceeded chance level, which is reflected in the intercept of the generalized linear mixed effects models (if performance is significantly above chance level, the CI does not contain the chance level probability associated with that task). As for the second research question, the model contained the between-participants predictor Group (control versus dyslexia). Following the structure of the online VSL model, the offline models further contained the orthogonally coded Triplet Set and Random Order as within-participant predictors, Age as an exploratory between-participants predictor and by-subject intercepts.

#### A-NADL task

We largely followed Lammertink et al. [63] in our analysis of the online RT measure of the A-NADL task. Filler trials (20% of trials) and incorrect responses and cases in which no response was given (7.6% of target and non-target trials) were removed prior to analysis.

As in the SRT task, learning during the exposure phase of the A-NADL is assessed as the difference between RTs to predictable input in rule blocks and RTs to pseudo-random input in the disruption block (research question one). Therefore, in order to find out whether we find evidence of learning during exposure, the linear mixed effects model fitted normalized RTs as a function of the within-participant predictor Block (i.e. rule blocks 3 and 5 versus disruption block 4). Block was orthogonally coded into two contrasts: the effect of learning (i.e. disruption block 4 coded as -^2^/_3_ vs. rule blocks 3 and 5 coded as +^1^/_3_ each) and the contrast between the two rule blocks (block 3 vs. block 5 coded as -^1^/_2_ and +^1^/_2_ respectively). The second research question, which pertains to the effect of group, was investigated through the interaction between our measure of learning and the between-participant predictor Group (control versus dyslexia). Several other predictors are considered, since these may influence the findings of the model. These included the within-participants predictor Target Type (i.e. target or non-target, coded as -^1^/_2_ and +^1^/_2_ respectively) and the between-participants counter-balancing factor Experiment Version (i.e. attending to *lut* coded as -^1^/_2_ vs. *mip* coded as +^1^/_2_). Finally, Age was included in the model as an exploratory between-participants predictor. The random effects structure included by-subject and by-item random intercepts and by-subject random slopes for Block and Target Type and by-item random slopes for Experiment Version. The random effect of item refers to the individual X-elements used in the familiarization phase of the A-NADL (*N* = 24). By-item random slopes for group and the interaction between experiment version and group were removed. Similarly, by-subject random slopes for the interaction between Block and Target Type were removed. This was done because these resulted in near-perfect correlations, which means that the model was overparameterized. This removal did not result in a decrease in the fit of the model (*χ*^*2*^ = 8.178, *df* = 18, *p* = .98).

The offline measure of the A-NADL task consisted of yes/no responses to individual items in the GJT, which were coded as 1 (endorsements) or 0 (rejections). The data that served as input to the generalized linear mixed effects model was thus the proportion of endorsements versus rejections (i.e. endorsement rates). No data was removed prior to analysis of the offline GJT.

Importantly, whether an item is endorsed or rejected does not yet inform us about learning, since accuracy depends on the grammaticality of the item (i.e. whether the item adheres to the A-NADL structure or not). To assess whether children showed evidence of learning in the offline measure of the A-NADL (research question one), the model estimated the within-participants effect of Grammaticality (grammatical vs. ungrammatical items orthogonally coded as +^1^/_2_ and -^1^/_2_ respectively) on endorsement rates. The interaction between Grammaticality and Group would provide evidence of a potential difference in performance between children with and without dyslexia (research question two). Since test items either tested familiar X-elements or generalization through novel X-elements, the within-participants predictor Generalization (no coded as -^1^/_2_ vs. yes coded as +^1^/_2_) was included in the model. Finally, following the online model of the A-NADL, the offline model contained the between-participant counter-balancing predictor Experiment Version and the exploratory between-participant predictor Age. By-subject and by-item random intercepts and by-subject random slopes for Grammaticality and Target Type and random slopes for Experiment Version were included in the random effects. As in the online measure of the A-NADL, the random effect of item refers to the individual X-elements used in the test phase of the A-NADL (*N* = 16). By-item random slopes for group and the interaction between experiment version and group and by-subject random slopes for the interaction between Block and Target Type were removed due to overparameterization. Importantly, the fit of the model did not decline (*χ*^*2*^ = 1.793, *df* = 11, *p* = .999). In order to compute the CIs and p-values of the final model, the effect of Grammaticality, which was non-significant, was removed from the random effects structure.

## Results

We focus on confirmatory analyses aimed at answering our research questions. Each time, we separately present results of some of the exploratory analyses, which are not related to our research questions but may nevertheless be interesting (cf. [87]).

Since multiple measures were used to answer our research questions in the VSL and A-NADL tasks, all CIs (and associated significance criteria for *p*-values) of confirmatory results were Bonferroni-corrected to keep the overall false detection rate at 0.05. In the VSL, we used four measures to assess learning (i.e. two online measures: the effect of element and the effect of element in interaction with time, and two offline measures: 2-AFC and 3-AFC accuracy) and thus CIs were corrected for quadruple testing (CIs thereby correspond to a false detection rate of 0.05 / 4 = 0.0125 for each effect, i.e. we have 98.75% CIs). CIs were corrected for double testing in the A-NADL (i.e. one online and one offline measure), resulting in 97.5% CIs.

As suggested by reviewers, supplementary analyses were conducted including the order of the SL tasks as described in the general procedure as an additional predictor (see OSF for *R* Markdown and html files containing supplementary analyses). Since task order did not interact with our measures of learning (all *t* and *z* values < 1.8) and did not result in three-way interactions with our measures of learning and group (all *t* and *z* values < 1.8), results from the three testing orders were collapsed in subsequent sections that describe the results of the SRT, VSL and A-NADL tasks.

### SRT task

**Fig 1:**
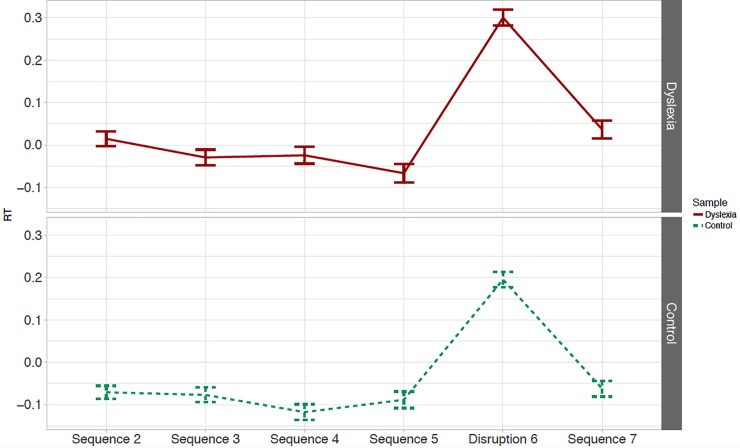
SRT task. Mean normalized RTs (+/- 1 SE) across blocks for participants with dyslexia (top graph; red solid line) and control participants (bottom graph; green dashed line).

Overall accuracy for both the TD (*M* = 93.8%) and dyslexia (*M* = 94.3%) groups was high, indicating that children attended to the task. Fig 1 presents the mean normalized RTs to accurate trials across the blocks of the SRT task. RTs were significantly shorter to structured input in sequence blocks 5 and 7 than to random input in disruption block 6 (*Δz* = -0.276, 95% CI [-0.329 … -0.223], *t* = -10.292, *p* = 7.5·10^−9^), indicating an effect of learning the SRT sequence in children when collapsing over groups. Group did not significantly influence the difference in RT to structured as opposed to unstructured input (*Δz* = -0.027, 95% CI [-0.133 … +0.079], *t* = -0.507, *p* = .61). In other words, there is no evidence for a difference in performance between children with and without dyslexia on the SRT task.

As mentioned, our model provides us with some exploratory findings. Firstly, no significant difference is found between RTs in the two structured blocks (*Δz* = +0.055, 95% CI [-0.003 … +0.113], *t* = 1.881, *p* = .063). The effect of group on the difference in RTs between the two structured blocks also does not reach significance (*Δz* = +0.073, 95% CI [-0.043 … +0.190], *t* = 1.248, *p* = .21). Although participants with dyslexia responded slightly slower than the control group overall, this effect does not reach significance (*Δz* = +0.102, 95% CI [-0.036 … +0.241], *t* = 1.462, *p* = .15). Participants’ age was found to influence RTs overall, with shorter RTs with increasing age (*Δz* = +0.102, 95% CI [-0.286 … +0.1448], *t* = -6.190, *p* = 7.5·10^−9^), but does not interact with the measure of learning and/or with group (see OSF). Lastly, adding attention and verbal short-term memory to the model does not change the main findings and does not significantly improve the model fit (*χ*^*2*^ = 5.410, *df* = 12, *p* = .94).

### VSL task

#### VSL task: Online RT measure

**Fig 2:**
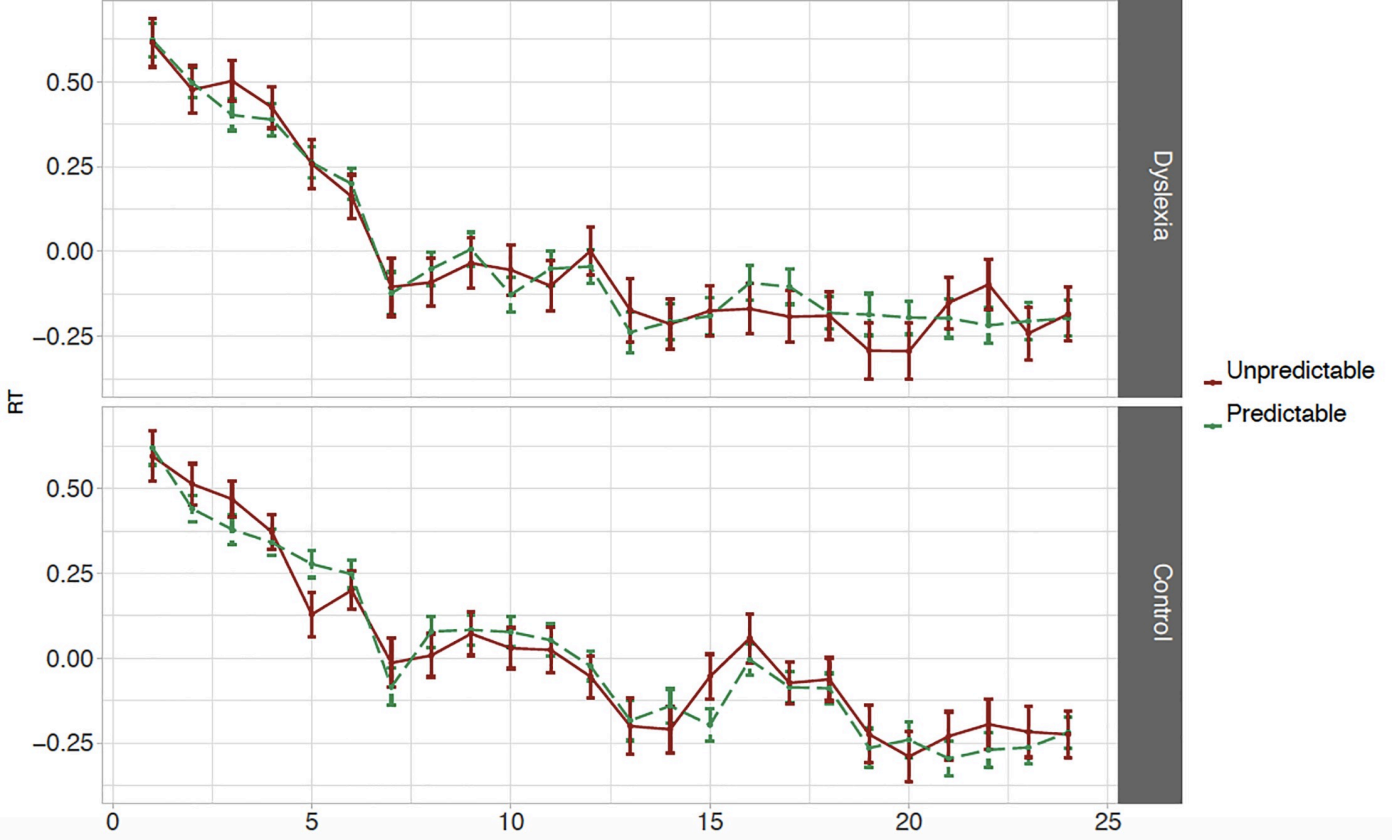
VSL task online RT measure. Mean normalized RTs (+/- 1 SE) to predictable (i.e. elements 2 and 3 within triplets; green dashed lines) and unpredictable (i.e. element 1; red solid lines) elements across repetitions of triplets during the exposure phase for participants with dyslexia (top graph) and control participants (bottom graph).

Responses to predictable elements were not significantly shorter as compared to unpredictable elements overall (Δ*z* = -0.013, 98.75% CI [-0.038 … +0.012], *t* = -1.271, *p* = .21) and there was no evidence of an effect of time in interaction with the online measure of learning (i.e. the difference between predictable and unpredictable elements; Δ*z* = -0.002, 98.75% CI [-0.024 … +0.019], *t* = -0.276, *p* = .78). Thus, we find no evidence of online sensitivity to the statistical structure in the VSL task. See Fig 2 for the mean normalized RTs to predictable and unpredictable elements across repetitions of triplets. The two-way interaction between the measure of learning and group (Δ*z* = +0.005, 98.75% CI [-0.038 … +0.047], *t* = 0.265 *p* = .79) and three-way interaction including time (Δ*z* = +0.024, 98.75% CI [-0.019 … +0.067], *t* = 1.404, *p* = .16) were both non-significant. We have no evidence that children with dyslexia perform the online VSL task differently than their TD peers.

The first exploratory finding is that participants with dyslexia responded slightly slower than participants in the control group, but this was not statistically significant (Δ*z* = 0.009, 95% CI [-0.250 … +0.267], *t* = 0.066, *p* = .95). Secondly, RTs were found to be significantly shorter to element 2 than to element 3 within triplets (Δ*z* = 0.045, 95% CI [+0.019 … +0.070], *t* = 3.501, *p* = .00057) and this effect was significantly larger in alien set A than in alien set B (Δ*z* = -0.190, 95% CI [-0.254 … -0.126], *t* = -5.889, 4.6·10^−9^). Note that there is no significant interaction between the difference in RTs to predictable elements and group (or alien set and group): there is no evidence that children with and without dyslexia perform differently with respect to the difference in RTs to predictable elements 2 and 3 (see OSF). Adding attention and verbal short-term memory to the model does not significantly improve the model fit (*χ*^*2*^ = 72.296, *df* = 96, *p* = .97) or influence the main findings regarding either research question.

#### VSL task: Offline 3-AFC and 2-AFC measures

**Fig 3:**
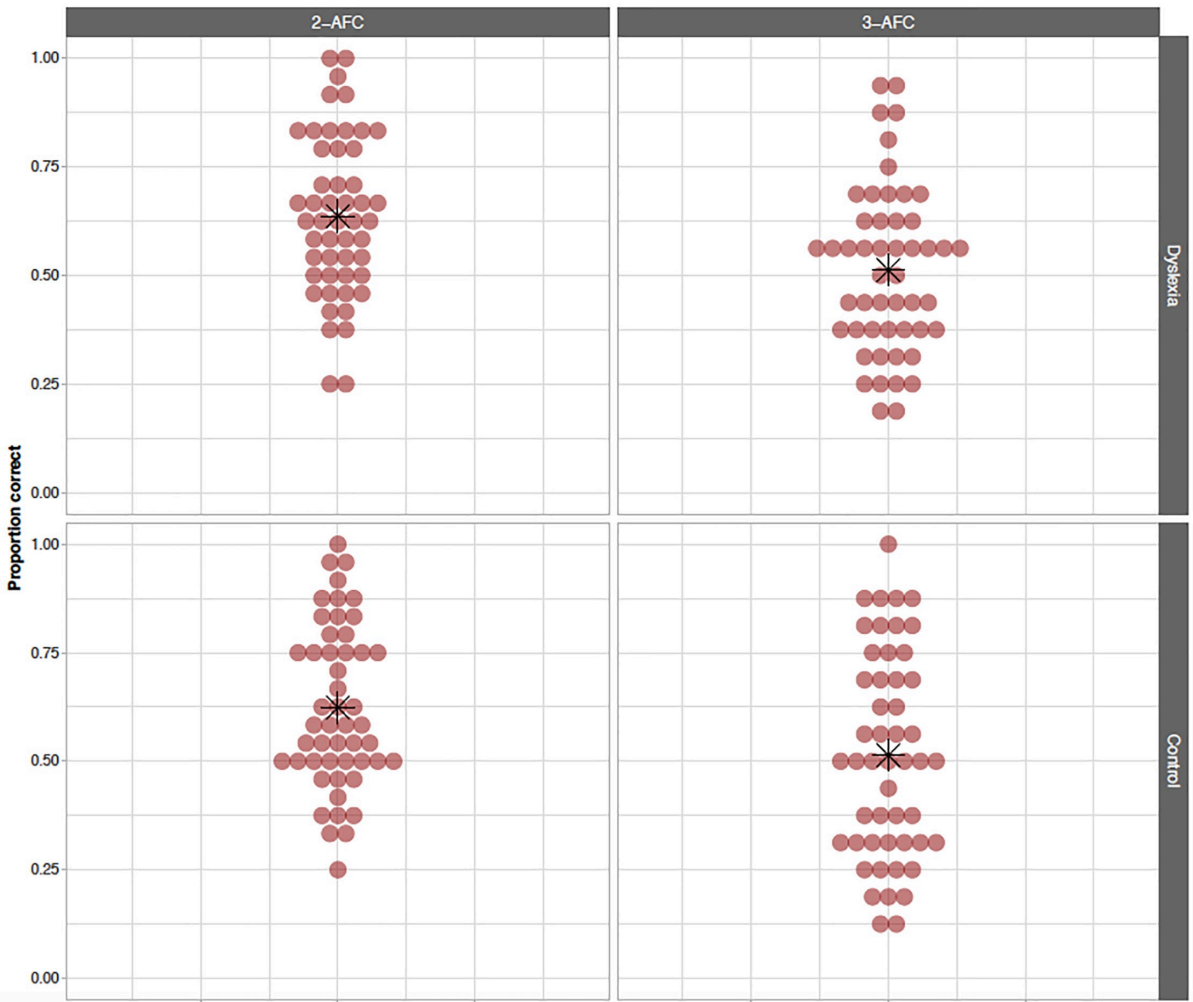
VSL offline 3-AFC and 2-AFC measures. Proportion of correct responses on 2-AFC (left, chance level = .500) and 3-AFC questions (right, chance level = .333) for participants with dyslexia (top) and control participants (bottom). Red dots indicate individual scores, while the group mean is indicated using a black asterisk.

Fig 3 presents the raw data of the offline test phase of the VSL. In our models, performance was estimated to be 18% and 15% above chance level in the 3-AFC and 2-AFC questions respectively, which was significant in both cases (3-AFC: probability estimate = .520, 98.75% CI = [.462 … .579], *p* = 1.8·10^−10^; 2-AFC: probability estimate = .653, 98.75% CI = [.600 … .704], *p* = 3.0·10^−10^). This means that, collapsing over group, children’s offline performance reveals learning in the VSL task. Pertaining to the second aim of our analysis, no significant effect of group was found on performance on 3-AFC (odds ratio estimate = 1.056, 98.75% CI = [0.659 … 1.695], *p* = .77) and 2-AFC (odds ratio estimate = 1.108, 98.75% CI = [0.701 … 1.751], *p* = .57) questions. Hence, there is no evidence that children with dyslexia perform the offline VSL tasks differently than their TD peers.

The first exploratory finding that should be noted is a significant interaction between alien set and group in the 3-AFC model (odds ratio estimate = 2.699, 95% CI = [1.298 … 5.662], *p* = .0084): participants with dyslexia performed better in alien set B than in alien set A, and the opposite pattern is observed in the control group. This interaction does not reach significance in the model of 2-AFC performance (odds ratio estimate = 1.839, 95% CI = [0.906 … 3.766], *p* = .091). Once again, adding attention and verbal short-term memory to the offline models does not significantly improve the model fit for either 3-AFC (*χ*^*2*^ = 18.242, *df* = 16, *p* = .31) or 2-AFC (*χ*^*2*^ = 21.884, *df* = 16, *p* = .15) questions and does not change the main findings of either model.

### A-NADL

#### A-NADL task: Online RT measure

**Fig 4:**
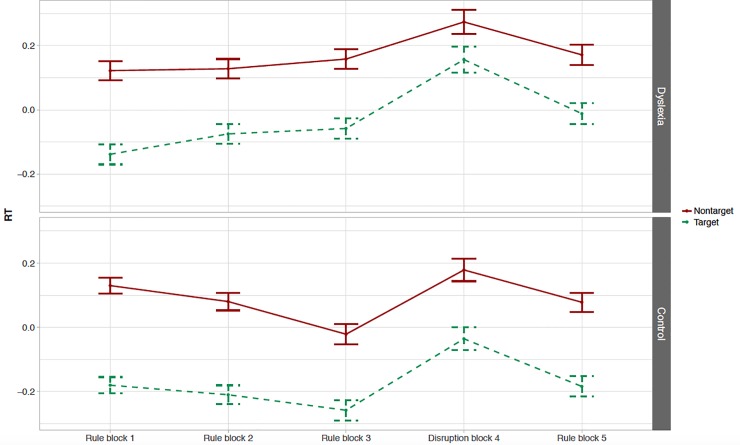
A-NADL online RT measure. Mean normalized RTs (+/- 1 SE) to nontarget (i.e. non-attended dependency; red solid line) and target (i.e. attended dependency; green dashed line) items across blocks for participants with dyslexia (top graph) and participants in the control group (bottom graph).

Overall accuracy during the online phase of the A-NADL was found to be high for both groups (TD: *M* = 95.5%, DD: *M* = 89.2%) indicating that participants attended to the task. Fig 4 presents the mean normalized RTs to targets and nontargets across the blocks of the A-NADL experiment. As predicted, RTs in rule blocks were significantly shorter than in the disruption block (Δ*z* = -0.159, 97.5% CI [-0.235 … -0.084], *t* = -4.796, *p* = 4.9·10^−6^). Thus, collapsing over group, we find evidence of online sensitivity to the NADL structure. There was no significant interaction between the effect of learning and group (Δ*z* = +0.011, 97.5% CI [-0.135 … +0.157], *t* = 0.167, *p* = .87). In other words, we find no evidence of a difference in online sensitivity to the A-NADL task between children with and without dyslexia.

Our model also provides us with an exploratory effect of target type, such that RTs were significantly shorter for the stimuli that the participants were attending to than those that were unattended (Δ*z* = +0.199, 95% CI [+0.156 … +0.242], *t* = 9.226, *p* = 2.7·10^−15^), and experiment version, such that RTs were shorter for participants who attended *lut* than for those that attended *mip* (Δ*z* = +0.176, 95% CI [+0.018 … +0.335], *t* = 2.197, *p* = .030). Additionally, target type and experiment version interact with one another and with our measure of learning (i.e. structured versus disruption blocks) in a three-way interaction (Δ*z* = +0.168, 95% CI [+0.014 … +0.322], *t* = 2.134, *p* = .033). Thus, we find evidence that the effect of learning is enhanced in targets vs. nontargets, especially when children received the version of the A-NADL where they were instructed to attend *lut*. Crucially, the main findings regarding our second research question are not influenced by these exploratory results: we find no significant interactions with the effect of group (see OSF). We found no significant difference in RTs to the two rule blocks included in analyses (i.e. rule block 3 vs. rule block 5; Δ*z* = +0.056, 95% CI [-0.012 … +0.125], *t* = 1.624, *p* = .11) and there was no significant interaction between this difference in RTs and group (Δ*z* = -0.050, 95% CI [-0.187 … +0.088], *t* = -0.718, *p* = .47). There is a marginally significant difference between participants with dyslexia and the TD participants in their overall RTs, with slower responses in the group of participants with dyslexia (Δ*z* = +0.152, 95% CI [-0.002 … +0.307], *t* = 1.950, *p* = .053). adding attention and verbal short-term memory to the model does not significantly improve the model fit (*χ*^*2*^ = 46.271, *df* = 48, *p* = .54) and does not influence the main findings of the online measure of the A-NADL task.

#### A-NADL task: Offline GJT measure

**Fig 5:**
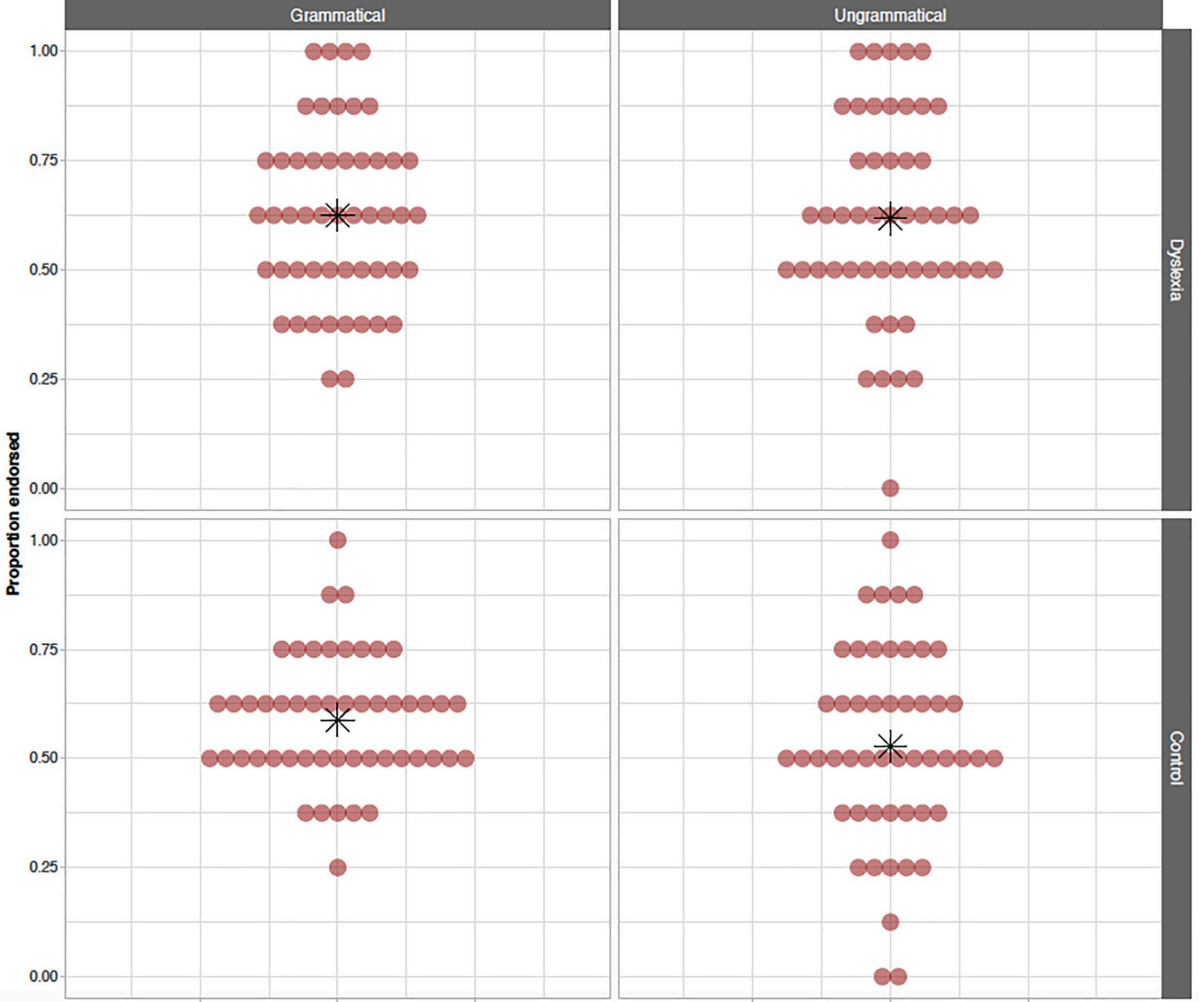
A-NADL offline GJT measure. Proportion of endorsements (i.e. proportion of items endorsed as opposed to rejected; chance level = .500) in the GJT for grammatical (left) and ungrammatical (right) items of participants with dyslexia (top) and control participants (bottom). Red dots indicate individual scores, while the group mean is indicated using a black asterisk.

Fig 5 presents the raw proportion of items endorsed (i.e. accepted versus rejected) for participants with and without dyslexia on both grammatical and ungrammatical items in the offline phase of the A-NADL task. The model estimated that the effect of grammaticality on endorsement rates did not reach significance (odds ratio estimate = 1.123, 97.5% Wald CI = [0.592 … 2.130], *p* = .68). Hence, we find no evidence of learning in children’s offline performance on the A-NADL. As for our second research question, we find no significant interaction between the effect of grammaticality and group (odds ratio estimate = .760, 97.5% Wald CI = [0.421 … 1.369], *p* = .30). Therefore, we find no evidence of a difference in performance on the offline measure of the A-NADL between children with and without dyslexia.

Besides these confirmatory findings, the model revealed a significant yes-bias in the offline measure of the A-NADL (odds estimate of the intercept = 1.507, 95% CI = [1.113 … 2.050], *p* = .011): children are more likely to endorse items as opposed to reject them overall. This effect was significantly larger in the group of participants with dyslexia as opposed to the participants in the control group (odds ratio estimate = 1.362, 95% CI = [1.016 … 1.840], *p* = .039), reflecting a larger yes-bias in children with dyslexia than in TD children. Secondly, we found that test items that contained a novel X-element were endorsed significantly less often than those that contained an X-element which had been heard during familiarization (odds ratio estimate = 0.335, 95% CI = [0.183 … 0.608], *p* = .0012). Again, this effect is significantly larger in participants with dyslexia when compared to the TD participants (odds ratio estimate = 1.916, 95% CI = [1.113 … 3.335], *p* = .019). We conclude that the endorsement preference (i.e. yes-bias) for familiar over novel X-elements is greater for children with dyslexia than for TD children. Note that these effects do not interact with our measures of learning (i.e. grammaticality) or the effect of group and thus do not influence the confirmatory results. As for our previous measures of SL, the findings regarding the offline measure of the A-NADL are not significantly affected by adding sustained attention and short-term memory to the model and the fit of the model is not significantly affected (*χ*^*2*^ = 44.369, *df* = 32, *p* = .072).

## Discussion

The present study investigated SL in children with dyslexia across three different experimental paradigms in a single sample, including the VSL and A-NADL paradigms that had not previously been used in child samples with dyslexia. We aimed to overcome raised methodological concerns as discussed in the introduction (e.g. [56,57,67]) by assessing learning through a range of online and offline measures and by controlling for group differences in underlying cognitive skills including memory and attention. Across the three SL tasks, we see the same pattern of results: we find evidence of learning when we collapse over groups and we find no evidence of a difference in performance between children with dyslexia and their TD peers. In all analyses, these results remain unchanged after controlling for individual differences in short-term memory and sustained attention. Similarly, the main findings across all SL measures are unaffected by participants’ age. Thus, this study finds no evidence in support of (or against) the idea that a (domain-general) SL deficit is the underlying cause of the literacy problems experienced by children with dyslexia. In the following sections we will elaborate on these findings and their implications.

### Measuring statistical learning in child populations

Although overall the same pattern of results arises such that 8- to 11-year-old children show (on- or offline) sensitivity to the statistical structures presented in the SRT, VSL and A-NADL tasks, the measures in the present study differed in their ability to detect learning in this age group. In the VSL task, children learned the structure as indicated by above chance performance on the offline 3-AFC and 2-AFC question, but the online measure did not reveal evidence of learning during the exposure phase. The fact that the children in the present study show offline learning is in line with previous studies that have indicated that, while offline tasks are problematic in younger school-aged children, performance increases between the ages of 5 and 12 [43,46]. Thus, for the VSL paradigm, the offline 3-AFC and 2-AFC questions used here have been demonstrated to be sensitive to learning in children between 8 and 11 years of age. However, we failed to replicate studies that suggested the added value of using the online RT measure of the self-paced VS in adults and children: participants in these studies responded more slowly to unpredictable stimuli than to predictable stimuli [60,62]. This failure to replicate could be due to small changes in the methodological design but may also be an indication that the RT measure of the VSL is not reliable enough to study performance in child populations. Since the observed difference in RTs between predictable and unpredictable elements across the experiment was deemed small in children between 5 and 8 years of age [62], this effect may be too small to reliably detect across studies and across samples. Future research should further investigate the usefulness of such an online measure and/or alternative online measures when studying SL through the VSL paradigm in children.

The A-NADL task revealed the reversed outcome: children were found to show online sensitivity to the nonadjacent structure, as indicated by an increase in RTs in the disruption block as opposed to RTs in the surrounding rule blocks, while there was no evidence that children endorse more grammatical than ungrammatical items in the offline test phase. This pattern of findings regarding the A-NADL replicates earlier findings in younger TD children by Lammertink et al. [63], who report online learning but null findings on 2-AFC questions in 5- to 8-year-olds. As suggested there, the insensitivity of offline tasks could be due to children’s difficulties with the meta-linguistic nature of this type of questions. The offline task used for the A-NADL in the present study, the GJT, could contribute to these difficulties, since we have evidence that children are more likely to endorse items than to reject them (i.e. yes-bias). Additionally, we found evidence that children are more likely to endorse items that contain a familiar X-element than an unfamiliar X-element regardless of their grammaticality. This suggests that children were focused on the X-element when answering ‘yes’ or ‘no’. More sensitive offline measures need to be developed to assess the outcome of the learning process in the A-NADL task by children. Importantly, however, the online measure has been shown to be a reliable measure of A-NADL in children, as we replicated the learning effect as reported by Lammertink et al. [63]. Therefore, future studies investigating A-NADL performance in children could adopt the online measure of learning (in addition to offline measures) to detect sensitivity to nonadjacent structures in speech during exposure.

### Statistical learning in dyslexia

The main aim of the present study was to elucidate the extent of the proposed SL difficulties in children with dyslexia. We did not find evidence of group differences on any of the on- or offline measures of the SRT, VSL or A-NADL tasks. Since these tasks assess SL across domains (visuo-motoric, visual and auditory respectively) and across different types of statistical structures (fixed sequence, adjacent and nonadjacent dependencies respectively), we can conclude that we find no support for (or against) a (domain-)general SL deficit in dyslexia.

Of course, a null result is difficult to interpret and can have many possible explanations beside the actual absence of the effect in reality and beside chance. To ascribe meaning to our findings, we have to show that the effects, if they exist at all, are small. Smallness of an effect can be measured by computing its maximal standardized effect size, i.e. by dividing the maximum absolute raw effect size (the greater absolute bound of the confidence interval) by the residual standard deviation of the relevant model. From these post-hoc effect size calculations, we obtain a maximal standardized effect size of 0.160/0.893 = 0.18 for the online SRT measure, for VSL we get 0.052/0.647 = 0.08, and for the A-NADL we get 0.168/0.815 = 0.21. Therefore, standardized effect sizes on the measures of all three tasks are below or around 0.20 and can therefore be called “small” [88]. One potential cause for the smallness of the effects could be that the selected subjects do not represent the average child with (or without) dyslexia. However, the children with and without dyslexia in the present study were carefully selected according to strict in- and exclusion criteria. The groups were not seen to differ from one another regarding their age, gender, SES and non-verbal reasoning, and the children with dyslexia showed impairments in tasks measuring reading, spelling and lexical retrieval as is characteristic of the disorder. Similarly, the difficulties with verbal short-term memory and sustained attention found in the present study have previously been reported in other samples of children with dyslexia [70–72]. These are indications that the group of participants with and without dyslexia are representative of the population as a whole. Another potential explanation for the smallness of the effects could be that the SL tasks used are not suitable to assess the underlying construct of SL. However, since we found evidence of learning overall in all three tasks, these paradigms are able to detect learning in children in this age group. Although the methodologies used to investigate SL in child populations should be improved to achieve a full picture of their SL abilities (i.e. the online measure in the VSL and offline measure in the A-NADL), the methodologies of the present study are sensitive enough to potentially detect group differences between participants with and without dyslexia. To summarize, it seems likely that 8- to 11-year-old children with dyslexia do not experience large problems with SL as assessed through these paradigms when compared to age-matched controls. Put more strongly, the results of the present study do not agree with the hypothesis that a domain-general deficit in SL underlies the literacy problems that we see in individuals with dyslexia.

Whereas these results may appear unexpected, other studies have also reported null results (without discussing the effect size) when investigating differences in SL performance between children with and without dyslexia on tasks tapping into SL abilities (SRT e.g. [34,36,41], AGL: e.g. [24,89]). Recently, authors have reached similar inconclusive results regarding the SL deficit hypothesis of dyslexia in literature reviews and meta-analyses of the SRT and AGL paradigms [28,29], because these studies underlined the mixed findings (i.e. some studies report significant group effects, while others do not) and established the presence of a publication bias in the field. Of course, methodological differences between studies may (partially) explain the fact that some studies report significant group effects while others do not, especially when the sought-after effect is likely to be small. As also argued by Schmalz et al. [90] and Elleman et al. [91], the relationship between performance on SL tasks and literacy skills (and thus dyslexia) may only appear under specific conditions. For example, the type of SL task used (e.g. its statistical structure, its modality), but also the selection of participant groups (e.g. their age, native language, or cultural differences such as differing dyslexia treatments) may influence findings of individual studies. Furthermore, West et al. [56] question the relationship between SL abilities and dyslexia (and related language learning impairments) based on the poor reliability of the SL tasks used (SRT, Hebb repetition, and contextual cueing) and the lack of correlations between the SL tasks and performance on tasks assessing language and literacy (see also [90]).

To conclude, the mixed pattern of findings in the field, and the smallness of the effects found here, suggest that the difference in performance on SL tasks between participants with and without dyslexia may be small and may only be detected under certain experimental conditions (see also [91,92] for dissociations between different SL tasks).

### Directions for future research

Although the present study detected learning in all three SL paradigms tested (ie. SRT, VSL, A-NADL), some measures were shown to be less reliable in detecting learning than others (i.e. online learning in the VSL, offline learning in the A-NADL). Future studies that aim to investigate SL performance in children in general, or the relationship between SL and dyslexia more specifically, should aim to develop tasks that are increasingly suitable for assessing SL abilities in child participants. Additionally, follow-up research using the SRT task could include an explicit offline test phase or consolidation and retention phases in order to gain a complete picture of SRT performance in children with and without dyslexia [93,94]. This adaptation would also allow for a closer comparison with other SL tasks including both on- and offline phases (e.g. VSL and A-NADL).

Since the potential group effect may be small and susceptible to methodological differences between studies, exact replications and large-scale (cross-linguistic and/or cross-cultural) studies are needed to elucidate whether individuals with dyslexia experience (domain-general) difficulties in the area of SL. Future studies could (a priori [87,95]) choose to conduct Bayesian analyses in order to potentially find support for the null hypothesis that SL abilities in children with and without dyslexia do not differ. As evidence accumulates, existing meta-analyses [27,28,29] could be updated to include recent and future findings to further clarify the clinical relevance of SL in relation to dyslexia and could be extended to further investigate the potential effects of methodological differences between studies (e.g. type of task used, modality tested, and the age or native language of participants).

## Conclusions

This study examined the performance of children with and without dyslexia on three experimental paradigms assessing SL abilities. Across the SRT, VSL and A-NADL paradigms we find that, taken together, children with and without dyslexia are sensitive to the statistical structures presented to them and we find no evidence of a difference in performance between the two groups. Moreover, the group effects reported on in the present study were found to be small. These findings do not support the hypothesis that a domain-general SL deficit results in the literary problems that are observed in individuals with dyslexia. Although future studies are needed to further investigate the direct contribution of SL abilities to literacy acquisition, both in typical and impaired populations, the clinical relevance of SL in relation to dyslexia is likely to be small.

## References

[1] Snowling MJ. *Dyslexia*. Malden MA: Blackwell; 2000.

[2] Gathercole SE, Alloway TP, Willis C, Adams AM. Working memory in children with reading disabilities. *Journal of Experimental Child Psychology*, 2006; 93(3): 265–281. 10.1016/j.jecp.2005.08.003 16293261

[3] Melby-Lervåg M, Lyster SA, Hulme C. Phonological skills and their role in learning to read: A meta-analytic review. *Psychological Bulletin*, 2012; 138(2): 322–352. 10.1037/a0026744 22250824

[4] Ramus F, Rosen S, Dakin SC, Day BL, Castellote JM, White S, et al Theories of developmental dyslexia: insights from a multiple case study of dyslexic adults. *Brain*, 2003; 126(4): 841–865. 12615643 10.1093/brain/awg076

[5] Snowling MJ. From language to reading and dyslexia 1. *Dyslexia*, 2001; 7(1): 37–46. 10.1002/dys.185 11305230

[6] Rispens J, Been P. Subject-verb agreement and phonological processing in developmental dyslexia and specific language impairment (SLI): A closer look. *International Journal of Language & Communication Disorders / Royal College of Speech & Language Therapists*, 2007; 42(3): 293–305. 10.1080/13682820600988777 17514543

[7] Waltzman D, Cairns H. Grammatical knowledge of third grade good and poor readers. *Applied Psycholinguistics*, 2000; 21(2): 263–284.

[8] Stein J, Walsh V. To see but not to read; the magnocellular theory of dyslexia. *Trends in Neurosciences*, 1997; 20(4): 147–152. 9106353 10.1016/s0166-2236(96)01005-3

[9] Tallal P. Improving language and literacy is a matter of time. *Nature Reviews Neuroscience*, 2004; 5(9): 721–728. 10.1038/nrn1499 15322530

[10] Facoetti A, Paganoni P, Lorusso ML. The spatial distribution of visual attention in developmental dyslexia. *Experimental Brain Research*, 2000; 132(4): 531–538. 10.1007/s002219900330 10912834

[11] Ramus F. Developmental dyslexia: Specific phonological deficit or general sensorimotor dysfunction? *Current Opinion in Neurobiology*, 2003; 13(2): 212–218. 12744976 10.1016/s0959-4388(03)00035-7

[12] Ramus F, Pidgeon E, Frith U. The relationship between motor control and phonology in dyslexic children. *Journal of Child Psychology and Psychiatry and Allied Disciplines*, 2003; 44(5): 712–722. 10.1111/1469-7610.00157 12831115

[13] Nicolson RI, Fawcett AJ. Procedural learning difficulties: Reuniting the developmental disorders? *Trends in Neurosciences*, 2007; 30(4): 135–141. 10.1016/j.tins.2007.02.003 17328970

[14] Nicolson RI, Fawcett AJ. Dyslexia, dysgraphia, procedural learning and the cerebellum. *Cortex*, 2011; 47(1): 117–127. 10.1016/j.cortex.2009.08.016 19818437

[15] Frost R, Armstrong BC, Siegelman N, Christiansen MH. Domain generality versus modality specificity: The paradox of statistical learning. *Trends in Cognitive Sciences*, 2015; 19(3): 117–125. 10.1016/j.tics.2014.12.010 25631249 PMC4348214

[16] Perruchet P, Pacton S. Implicit learning and statistical learning: One phenomenon, two approaches. *Trends in Cognitive Sciences*, 2006; 10(5): 233–238. 10.1016/j.tics.2006.03.006 16616590

[17] Arciuli J. Reading as statistical learning. *Language, Speech, and Hearing Services in Schools*, 2018; 49(3S): 634–643. 10.1044/2018_LSHSS-STLT1-17-0135 30120442

[18] Arciuli J, Simpson IC. Statistical learning is related to reading ability in children and adults. *Cognitive Science*, 2012; 36(2): 286–304. 10.1111/j.1551-6709.2011.01200.x 21974775

[19] Frost R, Siegelman N, Narkiss A, Afek L. What predicts successful literacy acquisition in a second language? *Psychological Science*, 2013; 24(7): 1243–1252. 10.1177/0956797612472207 23698615 PMC3713085

[20] Jiménez-Fernández G, Vaquero JMM, Jiménez L, Defior S. Dyslexic children show deficits in implicit sequence learning, but not in explicit sequence learning or contextual cueing. *Annals of Dyslexia*, 2011; 61(1): 85–110. 10.1007/s11881-010-0048-3 21082295

[21] Vicari S, Marotta L, Menghini D, Molinari M, Petrosini L. Implicit learning deficit in children with developmental dyslexia. *Neuropsychologia*, 2003; 41(1): 108–114. 12427569 10.1016/s0028-3932(02)00082-9

[22] Gabay Y, Thiessen E D, Holt LL. Impaired statistical learning in developmental dyslexia. *Journal of Speech*, *Language*, *and* *Hearing Research*, 2015; 58(3): 934–945. 10.1044/2015_JSLHR-L-14-0324 PMC4490081 25860795

[23] Pavlidou EV, Williams JM. Implicit learning and reading: Insights from typical children and children with developmental dyslexia using the artificial grammar learning (AGL) paradigm. *Research in Developmental Disabilities*, 2014; 35(7): 1457–1472. 10.1016/j.ridd.2014.03.040 24751907

[24] Rüsseler J, Gerth I, Münte TF. Implicit learning is intact in adult developmental dyslexic readers: Evidence from the serial reaction time task and artificial grammar learning. *Journal of Clinical and Experimental Neuropsychology*, 2006; 28(5): 808–827. 10.1080/13803390591001007 16723326

[25] Inácio F, Faísca L, Forkstam C, Araújo S, Bramão I, Reis A, et al Implicit sequence learning is preserved in dyslexic children. *Annals of dyslexia*, 2018; 68(1): 1–14. 10.1007/s11881-018-0158-x 29616459

[26] Roodenrys S, Dunn N. Unimpaired implicit learning in children with developmental dyslexia. *Dyslexia*, 2008; 14(1): 1–15. 10.1002/dys.340 17624907

[27] Lum JAG, Ullman MT, Conti-Ramsden G. Procedural learning is impaired in dyslexia: Evidence from a meta-analysis of serial reaction time studies. *Research in Developmental Disabilities*, 2013; 34(10): 3460–3476. 10.1016/j.ridd.2013.07.017 23920029 PMC3784964

[28] Schmalz X, Altoè G, Mulatti C. Statistical learning and dyslexia: A systematic review. *Annals of Dyslexia*, 2017; 67(2): 147–162. 10.1007/s11881-016-0136-0 27766563

[29] van Witteloostuijn M, Boersma P, Wijnen F, Rispens J. Visual artificial grammar learning in dyslexia: A meta-analysis. *Research in Developmental Disabilities*, 2017; 70: 126–137. 10.1016/j.ridd.2017.09.006 28934698

[30] Plante E, Gómez RL. Learning without trying: The clinical relevance of statistical learning. *Language, Speech, and Hearing Services in Schools*, 2018; 49(3S): 710–722. 10.1044/2018_LSHSS-STLT1-17-0131 30120448 PMC6198914

[31] Nissen MJ, Bullemer P. Attentional requirements of learning: Evidence from performance measures. *Cognitive Psychology*, 1987; 19(1): 1–32.

[32] Lum J, Kidd E, Davis S, Conti-Ramsden G. Longitudinal study of declarative and procedural memory in primary school-aged children. *Australian Journal of Psychology*, 2010; 62(3): 139–148.

[33] Kidd E. Implicit statistical learning is directly associated with the acquisition of syntax. *Developmental Psychology*, 2012; 48(1): 171–184. 10.1037/a0025405 21967562

[34] Menghini D, Finzi A, Benassi M, Bolzani R, Facoetti A, Giovagnoli S, et al Different underlying neurocognitive deficits in developmental dyslexia: A comparative study. *Neuropsychologia*, 2010; 48(4): 863–872. 10.1016/j.neuropsychologia.2009.11.003 19909762

[35] Laasonen M, Väre J, Oksanen-Hennah H, Leppämäki S, Tani P, Harno H, et al Project DyAdd: Implicit learning in adult dyslexia and ADHD. *Annals of Dyslexia*, 2014; 64(1): 1–33. 10.1007/s11881-013-0083-y 24162872

[36] Deroost N, Zeischka P, Coomans D, Bouazza S, Depessemier P, & Soetens E. Intact first- and second-order implicit sequence learning in secondary-school-aged children with developmental dyslexia. *Journal of Clinical and Experimental Neuropsychology*, 2010; 32(6): 561–572. 10.1080/13803390903313556 19859852

[37] Waber DP, Marcus DJ, Forbes PW, Bellinger DC, Weiler MD, Sorensen LG, et al Motor sequence learning and reading ability: Is poor reading associated with sequencing deficits? *Journal of Experimental Child Psychology*, 2003; 84(4): 338–354. 12711531 10.1016/s0022-0965(03)00030-4

[38] Bussy G, Krifi-Papoz S, Vieville L, Frenay C, Curie A, Rousselle C, et al Apprentissage procédural implicite dans la dyslexie de surface et la dyslexie phonologique. *Revue de Neuropsychologie*, 2011; 3(3): 141–146.

[39] He X, Tong SX. Quantity Matters: children with dyslexia are impaired in a small, but not large, number of exposures during implicit repeated sequence learning. *American Journal of Speech-Language Pathology*, 2017; 26(4): 1080–1091. 10.1044/2017_AJSLP-15-0190 28796861

[40] Kelly SW, Griffiths S, Frith U. Evidence for implicit sequence learning in dyslexia. *Dyslexia*, 2002; 8(1): 43–52. 10.1002/dys.208 11990224

[41] Staels E, Van den Broeck W. A specific implicit sequence learning deficit as an underlying cause of dyslexia? Investigating the role of attention in implicit learning tasks. Neuropsychology, 2017; 31(4): 371 10.1037/neu0000348 28150963

[42] Turk-Browne NB, Jungé JA, Scholl BJ. The automaticity of visual statistical learning. *Journal of Experimental Psychology*: *General*, 2005; 134(4): 552–564. 16316291 10.1037/0096-3445.134.4.552

[43] Arciuli J, Simpson IC. Statistical learning in typically developing children: The role of age and speed of stimulus presentation. *Developmental Science*, 2011; 14(3): 464–473. 10.1111/j.1467-7687.2009.00937.x 21477186

[44] Saffran JR, Aslin RN, Newport EL. Statistical learning by 8-month-old infants. *Science*, 1996; 274(5294): 1926–1928. 10.1126/science.274.5294.1926 8943209

[45] Siegelman N, Frost R. Statistical learning as an individual ability: Theoretical perspectives and empirical evidence. *Journal of Memory and Language*, 2015; 81: 105–120. 10.1016/j.jml.2015.02.001 25821343 PMC4371530

[46] Raviv L, Arnon I. The developmental trajectory of children’s auditory and visual statistical learning abilities: Modality-based differences in the effect of age. *Developmental Science*, 2017; e12593 10.1111/desc.12593 28901038

[47] Kirkham NZ, Slemmer JA, Johnson SP. Visual statistical learning in infancy: Evidence for a domain general learning mechanism. *Cognition*, 2002; 83(2): B35–B42. 11869728 10.1016/s0010-0277(02)00004-5

[48] Saffran JR, Johnson EK, Aslin RN, Newport EL. Statistical learning of tone sequences by human infants and adults. *Cognition*, 1999; 70(1): 27–52. 10193055 10.1016/s0010-0277(98)00075-4

[49] Sigurdardottir HM, Danielsdottir HB, Gudmundsdottir M, Hjartarson KH, Thorarinsdottir EA, Kristjánsson Á. Problems with visual statistical learning in developmental dyslexia. *Scientific Reports*, 2017; 7(1): 606 10.1038/s41598-017-00554-5 28377626 PMC5428689

[50] Singh S, Walk AM, Conway CM. Atypical predictive processing during visual statistical learning in children with developmental dyslexia: an event-related potential study. *Annals of Dyslexia*, 2018; 1–15. 10.1007/s11881-018-0158-x 29907920 PMC6390967

[51] Gómez RL. Variability and detection of invariant structure. *Psychological Science*, 2002; 13(5): 431–436. 10.1111/1467-9280.00476 12219809

[52] Grama IC, Kerkhoff A, Wijnen F. Gleaning structure from sound: The role of prosodic contrast in learning non-adjacent dependencies. *Journal of Psycholinguistic Research*, 2016; 45(6): 1427–1449. 10.1007/s10936-016-9412-8 26861215 PMC5093218

[53] Iao LS, Ng LY, Wong AMY, Lee OT. Nonadjacent dependency learning in Cantonese-speaking children with and without a history of specific language impairment. *Journal of Speech, Language, and Hearing Research*, 2017; 60(3): 694–700. 10.1044/2016_JSLHR-L-15-0232 28265645

[54] Kerkhoff A, De Bree E, De Klerk M, Wijnen F. Non-adjacent dependency learning in infants at familial risk of dyslexia. *Journal of Child Language*, 2013; 40(1): 11–28. 10.1017/S0305000912000098 23217289

[55] Kerkhoff A, de Bree E, Wijnen F. Can poor readers be good learners? in *Developmental Perspectives in Written Language and Literacy: In honor of Ludo Verhoeven*, 2017: 315–331.

[56] West G, Vadillo MA, Shanks DR, Hulme C. The procedural learning deficit hypothesis of language learning disorders: We see some problems. *Developmental Science*, 2018; 21(2): e12552. 10.1111/desc.12552 PMC5888158 28256101

[57] Arciuli J, Conway CM. The Promise—and Challenge—of Statistical Learning for Elucidating Atypical Language Development. *Current Directions in Psychological Science*, 2018, 1–9. 10.1177/0963721418779977 PMC6287249 30587882

[58] Misyak JB, Christiansen MH, Tomblin JB. On-line individual differences in statistical learning predict language processing. *Frontiers in Psychology*, 2010; 1: 31 10.3389/fpsyg.2010.00031 21833201 PMC3153750

[59] Siegelman N, Bogaerts L, Christiansen MH, Frost R. Towards a theory of individual differences in statistical learning. *Philosophical Transactions of the Royal Society B*, 2017; 372(1711): 20160059. 10.1098/rstb.2016.0059 PMC5124084 27872377

[60] Siegelman N, Bogaerts L, Kronenfeld O, Frost R. Redefining “learning” in statistical learning: What does an online measure reveal about the assimilation of visual regularities? *Cognitive Science*, 2017; 1–36. 10.1111/cogs.12556 PMC5889756 28986971

[61] Kidd E, Donnelly S, Christiansen MH. Individual differences in language acquisition and processing. *Trends in Cognitive Sciences*, 2017; 22(2): 154–169. 10.1016/j.tics.2017.11.006 29277256

[62] van Witteloostuijn M, Lammertink I, Boersma P, Wijnen F, Rispens J. Assessing visual statistical learning in early-school-aged children: The usefulness of an online reaction time measure. *Frontiers in Psychology*, under review. 10.3389/fpsyg.2019.02051 PMC6753232 31572261

[63] Lammertink I, van Witteloostuijn M, Boersma P, Wijnen F, Rispens J. Auditory statistical learning in children: Novel insights from an online measure. *Applied Psycholinguistics*, 2018; 1–24.

[64] López-Barroso D, Cucurell D, Rodríguez-Fornells A, de Diego-Balaguer R. Attentional effects on rule extraction and consolidation from speech. *Cognition*, 2016; 152: 61–69. 10.1016/j.cognition.2016.03.016 27031495 PMC4869066

[65] Baker CI, Olson CR, Behrmann M. Role of attention and perceptual grouping in visual statistical learning. *Psychological Science*, 2004; 15(7): 460–466. 10.1111/j.0956-7976.2004.00702.x 15200630

[66] Toro JM, Sinnett S, Soto-Faraco S. Speech segmentation by statistical learning depends on attention. *Cognition*, 2005; 97(2): B25–B34. 10.1016/j.cognition.2005.01.006 16226557

[67] Arciuli J. The multi-component nature of statistical learning. *Philosophical Transactions of the Royal Society B: Biological Sciences*, 2017; 372(1711): 20160058. 10.1098/rstb.2016.0058 PMC5124083 27872376

[68] Janacsek K, Nemeth D. The puzzle is complicated: When should working memory be related to implicit sequence learning, and when should it not? (Response to Martini et al.). *Cortex*, 2015; 64: 411–412. 10.1016/j.cortex.2014.07.020 25239854

[69] Lum JA, Conti-Ramsden G, Page D, Ullman MT. Working, declarative and procedural memory in specific language impairment. *Cortex*, 2012; 48(9): 1138–1154. 10.1016/j.cortex.2011.06.001 21774923 PMC3664921

[70] Buchholz J, Davies AA. Adults with dyslexia demonstrate space-based and object-based covert attention deficits: Shifting attention to the periphery and shifting attention between objects in the left visual field. *Brain and Cognition*, 2005; 57(1): 30–34. 10.1016/j.bandc.2004.08.017 15629211

[71] Bosse ML, Tainturier MJ, Valdois S. Developmental dyslexia: The visual attention span deficit hypothesis. *Cognition*, 2007; 104(2): 198–230. 10.1016/j.cognition.2006.05.009 16859667

[72] Cowan N, Hogan TP, Alt M, Green S, Cabbage KL, Brinkley S, et al Short‐term memory in childhood dyslexia: Deficient serial order in multiple modalities. *Dyslexia*, 2017; 23(3): 209–233. 10.1002/dys.1557 28497530 PMC5540735

[73] R Development Core Team. R: A language and environment for statistical computing. R Foundation for Statistical Computing, Vienna, Austria; 2008 ISBN 3-900051-07-0, URL http://www.R-project.org.

[74] Raven J. Raven progressive matrices. In *Handbook of nonverbal assessment*, 223–237. Springer, Boston, MA; 2003.

[75] Brus B, Voeten M. Eén Minuut Test. Vorm A en B. Schoolvorderingenvoor het lezen, bestemd voor het tweede t/m het vijfde leerjaar van de lagere school. Nijmegen: Berkhout Testmateriaal; 1972.

[76] van den Bos KP, Spelberg H, Scheepsma A, De Vries J. De Klepel. Vorm A en B. Een test voor de leesvaardigheid van pseudowoorden. Verantwoording, handleiding, diagnostiek en behandeling. Berkhout, Nijmegen, The Netherlands; 1994.

[77] Braams T, de Vos T. *Schoolvaardigheidstoets Spelling*. Amsterdam: Boom uitgevers Amsterdam; 2015.

[78] van den Bos KP, Lutje Spelberg HC. *Continu Benoemen & Woorden Lezen*. Amsterdam: Boom uitgevers Amsterdam; 2007.

[79] Kort W, Schittekatte M, Compaan E. *Clinical Evaluation of Language Fundamentals-4-NL*. Amsterdam: Pearson; 2008.

[80] Alloway TP. *Alloway Working Memory Assessment (AWMA)*. London: Pearson; 2012.

[81] Schittekatte M, Groenvynck H, Fontaine J, Dekker P. *TEAch: Test of Everyday Attention for Children*. Amsterdam: Pearson; 2007.

[82] Psychology Software Tools, Inc. [E-Prime 2.0]. Retrieved from http://www.pstnet.com; 2012.

[83] Schneider W, Eschman A, Zuccolotto A. E-Prime User's Guide. Pittsburgh: Psychology Software Tools, Inc; 2012.

[84] Bates D, Maechler M, Bolker B, Walker S. lme4: Linear mixed-effects models using Eigen and S4. R package version 1.1–7; 2014.

[85] Barr DJ, Levy R, Scheepers C, Tily HJ. Random effects structure for confirmatory hypothesis testing: Keep it maximal. *Journal of Memory and Language*, 2013; 68(3): 255–278. 10.1016/j.jml.2012.11.001 PMC3881361 24403724

[86] Baguley T. *Serious stats: A guide to advanced statistics for the behavioral sciences*. Macmillan International Higher Education, 2012; New York, United States.

[87] Wagenmakers EJ, Wetzels R, Borsboom D, van der Maas HL, Kievit RA. An agenda for purely confirmatory research. *Perspectives on Psychological Science*, 2012; 7(6): 632–638. 10.1177/1745691612463078 26168122

[88] Cohen J. Statistical power analysis for the behavioral sciences. Second edition Hillsdale, NJ: Erlbaum; 1988.

[89] Nigro L, Jiménez-Fernández G, Simpson IC, Defior Citoler S. Implicit learning of non-linguistic and linguistic regularities in children with dyslexia. *Annals of Dyslexia*, 2015; 1–17. 10.1007/s11881-015-0096-9 26494638

[90] Schmalz X, Moll K, Mulatti C, Schulte-Körne G. Is statistical learning ability related to reading ability, and if so, why? *Scientific Studies of Reading*, 2018; 1–13. 10.1080/10888438.2018.1549045 30718941 PMC6358202

[91] Gabay Y, Schiff R, Vakil E. Dissociation between the procedural learning of letter names and motor sequences in developmental dyslexia. *Neuropsychologia*, 2012; 50(10): 2435–2441. 10.1016/j.neuropsychologia.2012.06.014 22750119

[92] Henderson LM, Warmington M. A sequence learning impairment in dyslexia? It depends on the task. *Research in Developmental Disabilities*, 2017; 60: 198–210. 10.1016/j.ridd.2016.11.002 27856107

[93] Vicari S, Finzi A, Menghini D, Marotta L, Baldj S, Petrosini L. Do children with developmental dyslexia have an implicit learning deficit?. *Journal of Neurology, Neurosurgery & Psychiatry*, 2005, 76(10), 1392–1397. 10.1136/jnnp.2004.061093 PMC1739378 16170083

[94] Hedenius M, Persson J, Alm PA, Ullman MT, Howard JH Jr, Howard DV, et al Impaired implicit sequence learning in children with developmental dyslexia. *Research in developmental disabilities*, 2013, 34(11), 3924–3935. 10.1016/j.ridd.2013.08.014 24021394

[95] Simmons JP, Nelson LD, Simonsohn U. False-positive psychology: Undisclosed flexibility in data collection and analysis allows presenting anything as significant. *Psychological Science*, 2011, 22(11), 1359–1366. 10.1177/0956797611417632 22006061

